# Taxonomic Position and Phylogeny of the Genus *Vargasiella* (Orchidaceae, Vandoideae) Based on Molecular and Morphological Evidence

**DOI:** 10.1371/journal.pone.0098472

**Published:** 2014-06-03

**Authors:** Dariusz L. Szlachetko, Marcin Górniak, Marta Kolanowska, Joanna Mytnik-Ejsmont, Agnieszka K. Kowalkowska, Piotr Rutkowski, Tomasz Koliński

**Affiliations:** 1 Department of Plant Taxonomy and Nature Conservation, The University of Gdańsk, Gdańsk, Poland; 2 Department of Molecular Evolution, The University of Gdańsk, Gdańsk, Poland; 3 Department of Plant Cytology and Embryology, The University of Gdańsk, Gdańsk, Poland; 4 Department of Immunology, The Medical University of Gdańsk, Gdańsk, Poland; University of Tartu, Estonia

## Abstract

Since the description of the Neotropical genus *Vargasiella* in 1952, its taxonomic position has remained unclear, mainly due to a lack of sufficient data. In this study, the taxonomic position of *Vargasiella* was revised based on the outcomes of macro- and micromorphological studies, analyses of selected molecular markers and ecological methods of niche distribution modeling. The phylogenetic relationships were inferred using three DNA markers: *mat*K, *trn*L-F and ITS sequences. The morphological studies included the analysis of macromorphological features of herbarium specimens as well as micromorphological examination of preserved flowers. The ecological niche modeling was applied to identify the distribution of the suitable niches of the studied taxa. The relationships between *Vargasiella* and most similar taxa remain unresolved based on the molecular analysis. The outcomes from the morphological studies indicated significant differences between *Vargasiella, Warrea* and *Warreopsis*. Moreover, a niche shift in response to changing climate after the last glacial maximum is observed in *Vargasiella*, while no substantial changes in the occupied habitats were identified in the other related taxa. The clocktree of the Zygopetaleae estimated from the *mat*K gene indicated that the most recent common ancestors of *Vargasiella*, *Warrea* and *Warreopsis* originated in the Miocene, while the divergence time for *Vargasiella* and *Warrea* was assessed at approximately 5.4 Ma ago. *Vargasiella* seems to be an outshoot of the main branch of evolution of the Zygopetaleae. It is noteworthy that the *Vargasiella-Warrea* dichotomy could have taken place later than the divergence of *Warreopsis* from the mutual lineage. The molecular analysis and morphological data suggest that *Vargasiella* and *Warrea* could have evolved from a common ancestor. Accumulation of morphological differences and acceleration of the evolution of *Vargasiella* were more intensive than in other Warreinae and this could probably be synchronized with adaptation to different climatic conditions.

## Introduction

**Figure 1:**
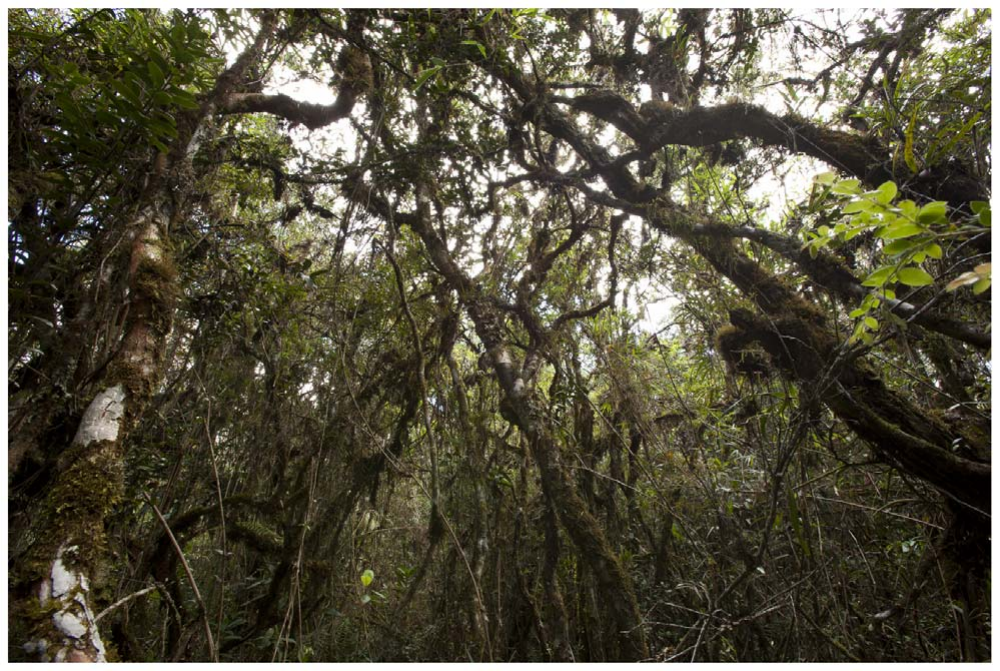
Habitat of *Vargasiella peruviana* C.Schweinf., Peru, Molinopampa (T. Kusibab).

**Figure 2:**
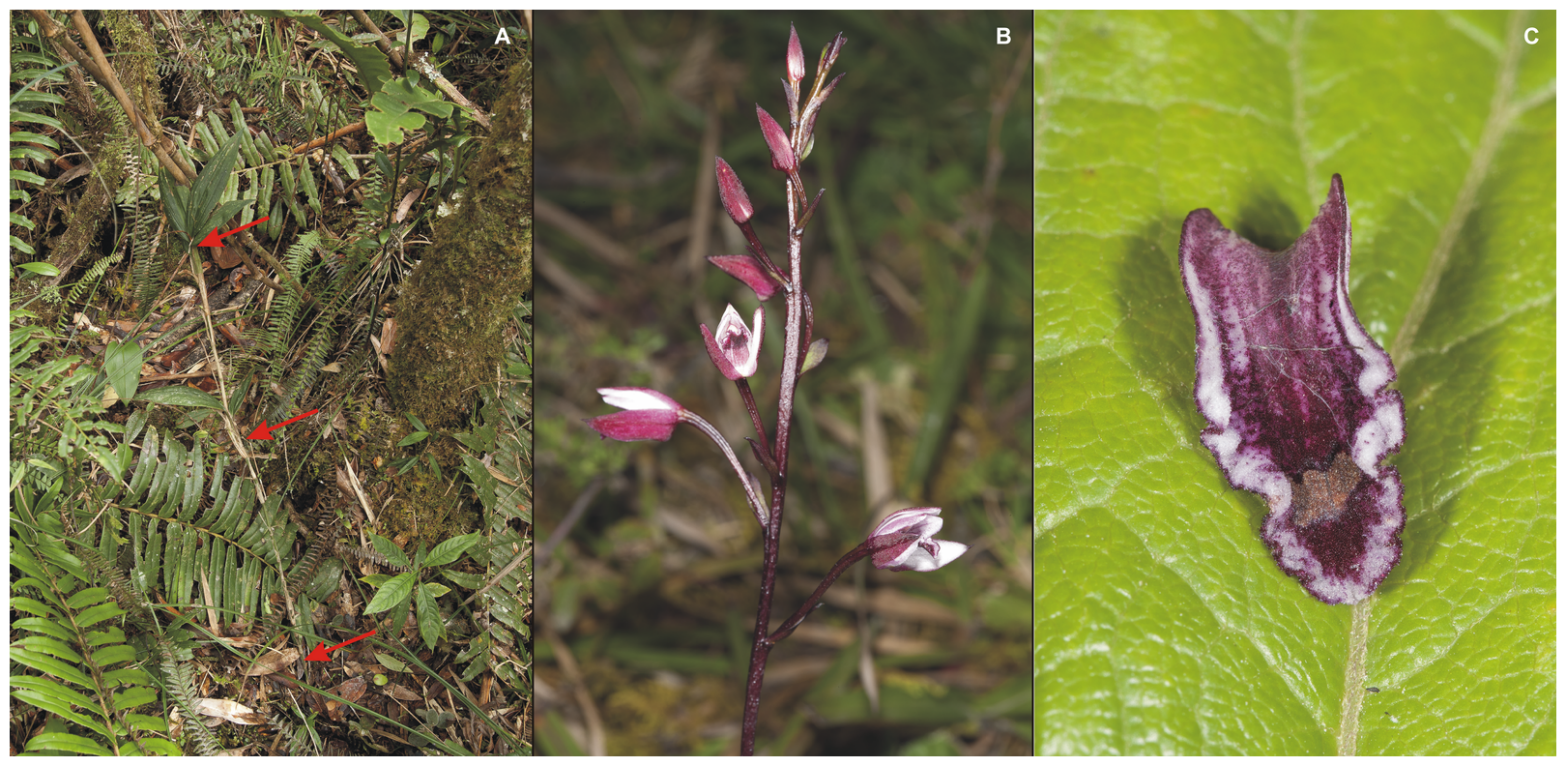
*Vargasiella peruviana* C.Schweinf. (A) Plant *in situ*; stem marked with red arrows. (B) Inflorescence. (C) Lip closeup. Photos: T. Kusibab.

The concept of the Neotropical orchid genus *Vargasiella* C.Schweinf. was proposed by Schweinfurth in 1952, along with the description of *Vargasiella peruviana* C. Schweinf., the only species known at that time. Six years later Schweinfurth [1] described another species, *Vargasiella venezuelana* C. Schweinf. Currently, the genus comprises only two species with a disjunctive distribution. They are terrestrial plants growing in wet, dense, submontane or montane forest, rich in epiphytes and lianas (Fig. 1). Populations of *Vargasiella peruviana* occasionally form quite large clumps in seasonally inundated meadows next to the forest edge. This plant's habit is unusual for orchids (Fig. 2a). Its stem is long, reaching about 1 m in length, creeping and climbing to nearby woody plants or robust grass, rooted occasionally. It appears to be monopodial which could be misleading; however, it becomes very clear that the stem consists of 15–25 cm-long segments if studied carefully. Each segment succeeds one arising from the apical part of the preceding one. The segments do not form any pseudobulbs, each of them is enclothed with 3–5 leaves. The leaf blades are oblong- or elliptic-lanceolate, convolute, thin-textured with 3–5 prominent, longitudinal nerves on the underside. The leaf blade is set on a short petiole, sheathed basally. The inflorescence is produced in the upper part of the segments (Fig. 2b). The peduncle is much longer than the laxly few-flowered raceme, the flowers are medium-sized and, in *V. peruviana*, rather attractive. The basic color is a mixture of deep purple and pink. The sepals and petals of *V. peruviana* are subsimilar, whereas in *V. venezuelana* the petals are wider than the sepals. The lip in both species is slightly different; in *V. peruviana* it is ovate-lanceolate to ligulate, undulate along upper margins with a thickened disc (Fig. 2c). The lip of *V. venezuelana* is elliptic-ovate to almost elliptic-orbicular, undulate above the cordate base, without any thickening.

The only other orchid genus that might possibly be confused with *Vargasiella* is *Palmorchis* Barb. Rodr.; however, this is possible only when exclusively vegetative characteristics are considered. Both genera are easily separable at the anthesis.

Since the description of the genus, the taxonomic position of *Vargasiella* has remained vague. Dressler [2] classified *Vargasiella* in his broadly defined tribe Maxillarieae Pfitzer, in the subtribe Zygopetalinae Schltr., into which he included a further 25 genera in four closely related alliances. He stated, however, that the relationship of monopodial *Vargasiella* to other genera of the subtribe is uncertain. Later, he suggested raising *Vargasiella* to subtribal rank [3], but upheld its position in the Maxillarieae. Simultaneously, he expressed doubts regarding the monopodial type of growth reported in that genus. Such a subtribe was validated in the same year by Romero and Carnevali [4]. In turn, Senghas [5] placed *Vargasiella* in the subtribe Liparidinae Lindl. *ex* Miq., based on the presence of naked pollinia which had already been reported by Schweinfurth [6], but without taking into consideration, however, the numerous differences in the gynostemium organization noted in these two taxa. In 1996, Senghas modified his opinion on that genus in response to Romero and Carnevali [4] who had noted the presence of tegula and viscidium in *Vargasiella peruviana.* Senghas [7] included the genus in the subtribal rank in the tribe Oncidieae Pfitzer, along with, inter alia, Dichaeinae Schltr., Pachyphyllinae Pfitzer, Ornithocephalinae Schltr. and Oncidiinae Benth. The combination of the gynostemium structure and the monopodial type of growth misled Szlachetko [8] into coming to the conclusion that Vargasiellinae, Dichaeinae and Pachyphyllinae might be cognate and he accepted the tribe Dichaeeae Pfitzer. Pridgeon et al. [9] included Vargasiellinae in the broadly defined Cymbidieae Pfitzer.

While the taxonomy of numerous orchid genera are still discussed and their systematic position is debated by scientists, especially when morphological data are in conflict with molecular analyses, the inconsistency in the classification of *Vargasiella* has mostly been related to the lack of material for genetic studies and the insufficient number of specimens for morphological examination. Both *Vargasiella* species have found growing in remote areas of Bolivia, Peru and Venezuela. Unfortunately, all efforts to keep them alive via cultivation have failed. During the scientific expeditions (2007, 2008) conducted to the Peruvian Departments of Loreto and Amazonas, we had the opportunity to study specimens of *Vargasiella peruviana in situ*. Based on macro- and micromorphological studies, analyses of selected molecular markers and ecological niche distribution modeling methods, we made an attempt to clarify the taxonomic placement of *Vargasiella*.

## Materials and Methods

### Field study

The population of *Vargasiella peruviana* has been found in the Peruvian District Molinopampa (Amazonas, Prov. Chachapoyas), about 25 km E of Chachapoyas, at the altitude of about 2400 m, in the mountainous area covered by patches of low forest separated by pasture and wet meadows. The plants have been found growing both inside and along edges of a forest. The standard measurements of vegetative parts and photographic documentation was taken in the field. The samples of the specimens collected in the Departments of Loreto and Amazonas for morphological and molecular analyses were collected thanks to the courtesy of Mr. Manolo Arias and his Peruana Orchid Nursery.

### Molecular study

#### DNA Isolation

Fresh leaf samples were preserved in silica gel. Total genomic DNA was extracted from 20 mg of dried leaves [11] using the DNA Mini Plant (A&A Biotechnology, Poland) following the manufacturer's protocol. Plant tissues were homogenized in a sterilized mortar and kept at −65°C for 12 hours.

#### Amplification

Nuclear ITS region (ITS1-5.8S-ITS2) was amplified by Polymerase Chain Reaction (PCR) method using the primers 17SE and 26SE [12] in a T1 thermocycler (Biometra, Germany). PCR was carried out in a volume of 50 µl. The PCR mixture contained: dd H_2_O, 5 µl 10x Polymerase buffer with 15 mM MgCl_2_, 2 µl of 5 mM mix of each dNTP (200 µM), 0.3 µl of 20 mM primers, 2 µl DMSO, 2.0 units of Taq DNA Polymerase (EURx, Gdańsk, Poland) and genomic DNA. The thermal cycling protocol of the PCR consisted of 5 min initial denaturation at 94°C, 28 cycles, each with 45s denaturation at 94°C, 45 s annealing at 53°C and an extension of 60 s at 72°C, ending with an extension of 5 min at 72°C.

The *mat*K gene (about 1300 bp) was amplified with the following two primers: - 19F [13] and 1326R [14]. The PCR mixture contained: dd H_2_O, 5 µl 10x Polymerase buffer with 15 mM MgCl_2_, 1 µl of 10 mM mix of each dNTP (200 µM), 0.3 µl of 20 mM primers, 2 µl MgCl_2_ [50 mM], 2.0 units of Taq DNA Polymerase and genomic DNA. The thermal cycling protocol comprised 28 cycles, each with 45 s denaturation at 94°C, 45 s annealing at 52°C, an extension of 2 min 30 s at 72°C, concluding with an extension of 5 min at 72°C.

The *trn*L-F region containing the *trn*L intron and the *trn*L-*trn*F intergenic spacer was amplified using primers *trn*L-c and *trn*L-F as described by Taberlet [15]. Polymerase chain reaction (PCR) amplifications were carried out in a total volume of 25 µl containing 5 µl 5x buffer, 1 µl 50 mM MgCl_2_, 1 µl 5 mM dNTPs, 0.5 µl of 10 µM of each primers, and 1.0 unit of Blue Perpetual DNA polymerase (EURx, Gdańsk, Poland). The thermal cycling protocol of the PCR consisted of 5 min initial denaturation at 80°C, 30 cycles, each with 60 s denaturation at 94°C, 60 s annealing at 51°C and an extension of 2 min at 65°C, ending with an extension of 7 min at 65°C. Amplified products were cleaned with High Pure PCR Product Purification Kit (Roche Diagnostic GmbH, Mannheim, Germany) following the manufacturer protocol.

#### Sequencing

Cycle sequencing was carried out directly on the purified product using a Big Dye Terminator v 3.1 Cycle Sequencing Kit (Applied Biosystems, Warrington, Cheshire, UK): 2 µl of sequencing buffer, 2 µl of Big Dye terminator with 2 µl of 0,08 mM primer (1.6 pmol), 2–4 µl of amplified product (30–90 ng/ µl), and dd H_2_O in a total reaction volume of 10 µl. The PCR primers were used to sequence both strands of ITS and *trn*L-F. Two PCR primers (-19F, 1326R) and one internal primer (390F) were used [14] to sequence the *mat*K gene. Cycle sequencing condition for two strands (all regions) were as follows: 20 s initial denaturation followed by 25 cycles each with 15 s denaturation (94°C), 5 s annealing (50°C) and 4 min elongation (60°C) using T1 thermocycler (Biometra, Germany). Sequencing reactions were purified with an ExTerminator Kit (A&A Biotechnology, Gdynia, Poland) following the manufacturer's protocol. Pelleted samples were sequenced on an Applied Biosystems 377 automated sequencer. Both strands were sequenced to enssure accuracy in base calling. A Sequence Navigator (Applied Biosystems) was used to edit the sequences and each individual base position was examined for agreement between the two strands using AutoAssembler (Applied Biosystems) software. The *mat*K, *trn*L-F (containing *trn*L intron and *trn*L-*trn*F intergenic spacer), and ITS1-5.8S-ITS2 (nuclear ribosomal DNA; referred to as ITS) sequences for *Vargasiella peruviana* were deposited in the International Nucleotide Sequence Databases (INSD) under accession numbers KF938587/KF938588/KF938586 respectively.

#### Phylogenetic analysis

To enlarge the dataset for all representatives of the subtribe Zygopetalinae, additional 84 sequences were taken from INSD. All sequences for *mat*K, *trn*L-F, and ITS are derived from the publication of Whitten [10] (INSD PopSet no. 58200482/580042546/580042630 respectively) except for *Warrea warreana* (AF239417 – *mat*K, AF239513 – *trn*L, AF239321 – ITS). The list of analysed sequences downloaded from INSD is provided in Annex S1. The DNA sequences were aligned by ClustalX [16] and adjusted by eye using Seaview [17]. Maximum parsimony analyses were undertaken using heuristic searches in PAUP* version beta 10 [18] with tree-bisection-reconnection (TBR) branch swapping and the MULTREES (holding multiple trees) option in effect with 1000 replicates of random sequence addition. Only 10 trees were saved for each replicate to reduce the time spent in swapping large numbers of suboptimal trees. All characteristics were treated as unordered and as equally weighted [19]. Tree length, consistency index (CI) and retention index (RI) were estimated. The internal support of clades was evaluated using non-parametric bootstrapping [20] with 1000 replicates and the same settings as above, except for the simple sequence addition. Incongruence between data sets was tested using the incongruence-length difference (ILD) test [21] using PAUP*.

### Time calibration

The *mat*K matrix was used to estimate the calibration point for MRCA (most recent common ancestor) of the *Warrea-Vargasiella* clade. The Bayesian uncorrelated relaxed molecular clock approach implemented in BEAST [22] was used. The age for the root of the tree was set to a normal prior distribution with a mean of 19 Ma and a standard deviation of 5.0 (giving a 95% CI ranging from *c*. 11–27 Ma) corresponding to the resulting age estimate for the *Oncidium-Lycaste* clade from a calibration based on the analysis of *mat*K+*rbc*L for Orchidaceae [23]. The Yule process was chosen as the speciation process for the *mat*K data matrix. The Akaike Information Criterion in a ModelTest v.3.7 [24] was used to choose the best-fitting evolutionary model for *mat*K (GTR+Γ+I). Runs were performed in BEAST with 20 million generations each. Log files were analysed with Tracer v1.5 [25]. All resulting trees were then combined with LogCombiner v1.7.3 [22], with a burn-in of 20%. A maximum credibility tree was then produced using TreeAnnotator v1.5.3 [22].

### Macromorphological features

The examined plant material was obtained from the AMES, MO, UGDA and USM herbaria. Moreover, the observations and remarks made during two scientific expeditions (2007, 2008) to the Peruvian departments of Loreto and Amazonas were included into the analysis. The herbarium material was prepared according to the standard classical taxonomy procedure and examined under a stereomicroscope. The morphological descriptions were based on the observation of the type and non-type specimens deposited at AMES and preserved in liquid (UGDA, USM). The following vegetative characters of individual plants were analysed: stem (height, type of growth), leaves (number, size, shape), sheaths (number, shape, size), inflorescence (size, density), floral bracts (shape), flowers taken from the middle part of the inflorescence (size and surface of pedicel and ovary, presence of mentum, size and shape of perianth parts), as well as gynostemium (size and shape of the column, presence of column foot). The most discriminative characters have been selected and used in both phenetic as well as cladistic analyses (Annex S2, Annex S3). Particular parts of the flower were softened in boiling water prior to dissection followed by stereomicroscope examination and drawing. The database of the line-drawings and photographs of all studied specimens is available in the first author's archives. A key for determination of the *Vargasiella* species is provided. The line drawings were prepared using CorelDRAW Graphics Suite 12.

### Macromorphological analyses

To create hierarchic phenograms and cladograms, the PAST program [26] was used. A distance matrix was created using the most common Euclidean measure and middle links rule unweighted pair-group average (UPGMA) as an amalgamation rule. Neighbor joining is a bottom-up clustering method for the creation of phylogenetic trees proposed by Saitou and Nei [27]. The principle of this method is to find pairs of taxa (neighbors) that minimize the total branch length at each stage of the clustering of taxa. Finally, a character-based cladistical method - maximum parsimony with Fitch maximum parsimony algorithm – was used, which rapidly and precisely evaluates the minimum number of changes. This method infers a phylogenetic tree by minimizing the total number of evolutionary steps required to explain a given set of data or, in other words, by minimizing the total tree length (Occam's razor). *Vargasiella* was treated as a output group and for searching for the optimal tree structure we used heuristic algorithm tree bisection and reconnection (TBR), that detaches a subtree from the main tree at an interior node and then attempts all possible connections between branches of the two trees thus created. For phylogenic estimation, we used a bootstrap calculation with 1000 sampling.

### Micromorphological study

Fresh flowers were gathered and preserved in Kew Mixture in the following proportions: 70% of 95% ethanol, 5% of glycerin, 5% of formaldehyde and 20% of distillate water. The plant material was examined using a Nikon SMZ 1500 light microscope with a Nikon Digital Sight DS-Fi1 camera. For observations in the scanning electron microscope (SEM) the samples kept in the Kew mixture were dehydrated through ethanol series. To preserve the surface structure of the specimens, the procedure of critical-point drying in an Emitech K850 Critical Point Dryer apparatus was applied prior to mounting the samples on SEM stubs with an SPI Carbon Conductive Double Sided Adhesive Disc. Then, the plant material was gold-coated (Sputter Coater Spi-Module). The samples were examined and imaged in a Phillips XL-30 Scanning Electron Microscope operating at an accelerating voltage of 15kV.

### Ecological niche modeling

The ecological niche modeling (ENM) was applied to locate the glacial refuge areas and to estimate the potential range of *Vargasiella* and *Warrea* based on the suitable habitat distribution. Because the biogeographic data about both studied taxa are poor, we decided to include *Warreopsis* into the ENM analysis as an additional datum. The list of localities used in the analysis was compiled based on the data taken from the herbarium specimens' labels complemented by information from the electronic database of Missouri Botanical Garden (available at www.tropicos.org). Only those localities which could be precisely placed on the map were used in the ecological niche modeling. Six locations of *Vargasiella*, 19 *Warrea* and 10 *Warreopsis* populations were used (Annex S4), which is more than the minimum number required to obtain reliable predictions in the MaxEnt application [28]. As input data of 19 climatic variables in 2.5 arc minutes (±21.62 km^2^ at the equator) developed by Hijmans and others [29] as well as the elevation data were used (Table S1). The bioclimatic data for the last glacial maximum (LGM, between 26500 and 19000–20000 years ago) were developed and mapped by Paleoclimate Modeling Intercomparison Project Phase II [30]. To the accuracy of the modeling, the maximum iterations was set to 10,000 and convergence threshold to 0.00001. For each run 20% of the data were used to be set aside as test points [31]. The "random seed" option which provided random test partition and background subset for each run was applied. Each run was performed as a bootstrap with 1000 sampling and the output was set to logistics. Models were evaluated using the threshold-independent area under the Receiver Operator Characteristic curve (AUC), which is a measure of discrimination. All operations on the GIS data were carried out on ArcGis 9.3 [32].

### Range and niche overlap

The overlapping of the ranges and niches calculated for LGM and present time were determined using ENMTools application. Since the ecological amplitude of the studied taxa seems to be narrow, the threshold used in the calculation of the range overlap was 0.8. The niche overlap was measured using Schoener's D (D) [33] and I statistic (I) [34]. Schoener's D was initially developed to compare diet and microhabitats and here it is used with the assumption that direct measures of local species density are comparable with each other. The I statistic is based on the Hellinger distance and measures the ability of the model to estimate the true suitability of a habitat. Both metrics range from 0 (niches completely different) to 1 (total overlap).

## Results

### Molecular analyses

**Figure 3:**
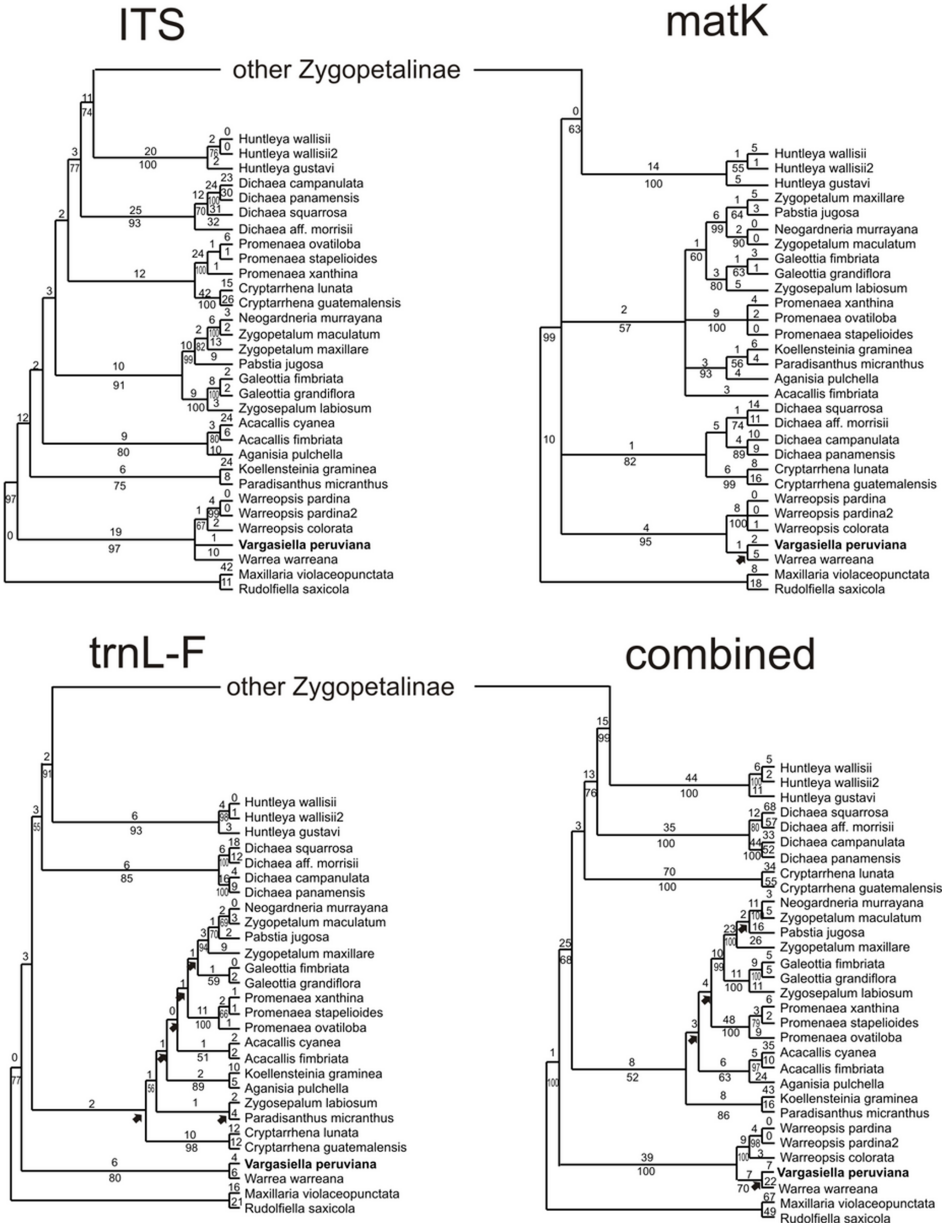
One of the most parsimonious trees in the ITS, *mat*K and *trn*L-F analyses for Zygopetalinae Schltr. The *Vargasiella peruviana* C.Schweinf. is highlighted in bold. The numbers above branches are Fitch branch lengths. Bootstrap percentages >50 are given for supported clades below branches. Arrows indicate clades that collapse in the strict consensus tree.

The result of the individual and combined analyses are presented in one of the most parsimonious trees. Bootstrap support (BS) above 50% is given for supported clades below branches. Branches that collapse in the strict consensus tree are indicated by arrows. Statistics for one of the most parsimonious trees from each analysis is shown in the Table 1. Statistics for *mat*K, *trn*L-F, ITS and combined data matrices are separated by “/”. The number of analysed taxa were 85/82/85/85 respectively. The aligned matrix comprised 1327/1389/842/3558 characters of which 233/286/374/1392 were variable and 134/132/263/529 were potentially parsimony informative. The number of the most parsimonious trees were 48/416/3258/10.000. Tree-length was 374/995/818/1640, consistency index (CI) = 0.72/0.77/0.77/0.68, and retention index (RI) = 0.83/0.86/0.86/84. Results from the *partition homogeneity test* indicate incongruence between all (plastid and nuclear partitions): *mat*K/*trn*L-F P = 0.01; *mat*K/ITS P = 0.01; *trn*L-F/ITS P = 0.01; *mat*K/*trn*L-F/ITS P = 0.01. A visual comparison of the topology and bootstrap support between plastid and ITS data sets shows incongruence between low supported clades. In this case combined analyses were performed. On a combined ITS-*mat*K-*trn*L-F tree (Fig. 3), the analysed genera are arranged in groups congruent with Szlachetko's subtribes [8], i.e. Huntleyinae Schltr. (pseudobulbs lacking or obscure), Zygopetalinae *s.str.* (heteroblastic pseudobulbs) and Warreinae Szlach. (homoblastic pseudobulbs). Dichaeinae (monopodial plants) and Cryptarrheninae Dressl. (pseudobulbs obscure or absent) are nested in this clade as well. In all analyses, *Vargasiella* composes a highly supported subclade together with *Warrea* and *Warreopsis* (ITS 97 BS; *mat*K 95 BS; *trn*L-F 80 BS (only *Warrea-Vargasiella*); combined 100 BS). The subclade is situated at the base of the Zygopetalinae *sensu lato* clade and it is sister to the rest of the taxa (except for the *mat*K tree where it forms a basal polytomy with the other subclades). The relationships between *Warrea, Warreopsis* and *Vargasiella* are not resolved. In all strict consensus trees (not shown) the *Warrea-Vargasiella* clade present in the *mat*K and combined trees is collapsed.

**Table 1:** Statistics for one of the most parsimonious trees from each analysis (CI - consistency index; RI - retention index).

matrix	*mat*K	trnL-F	ITS	*mat*K/trnL-F/ITS
No. of taxa	85	82	85	85
Included positions in matrix	1327	1389	842	3558
Variable site	233	286	374	1392
Parsimony-informative sites	134	132	263	529
Trees (MPT)	48	416	3258	10000
Fitch tree length	374	995	818	1640
CI	0.72	0.77	0.77	0.68
RI	0.83	0.86	0.86	0.84

### Tree calibration

**Figure 4:**
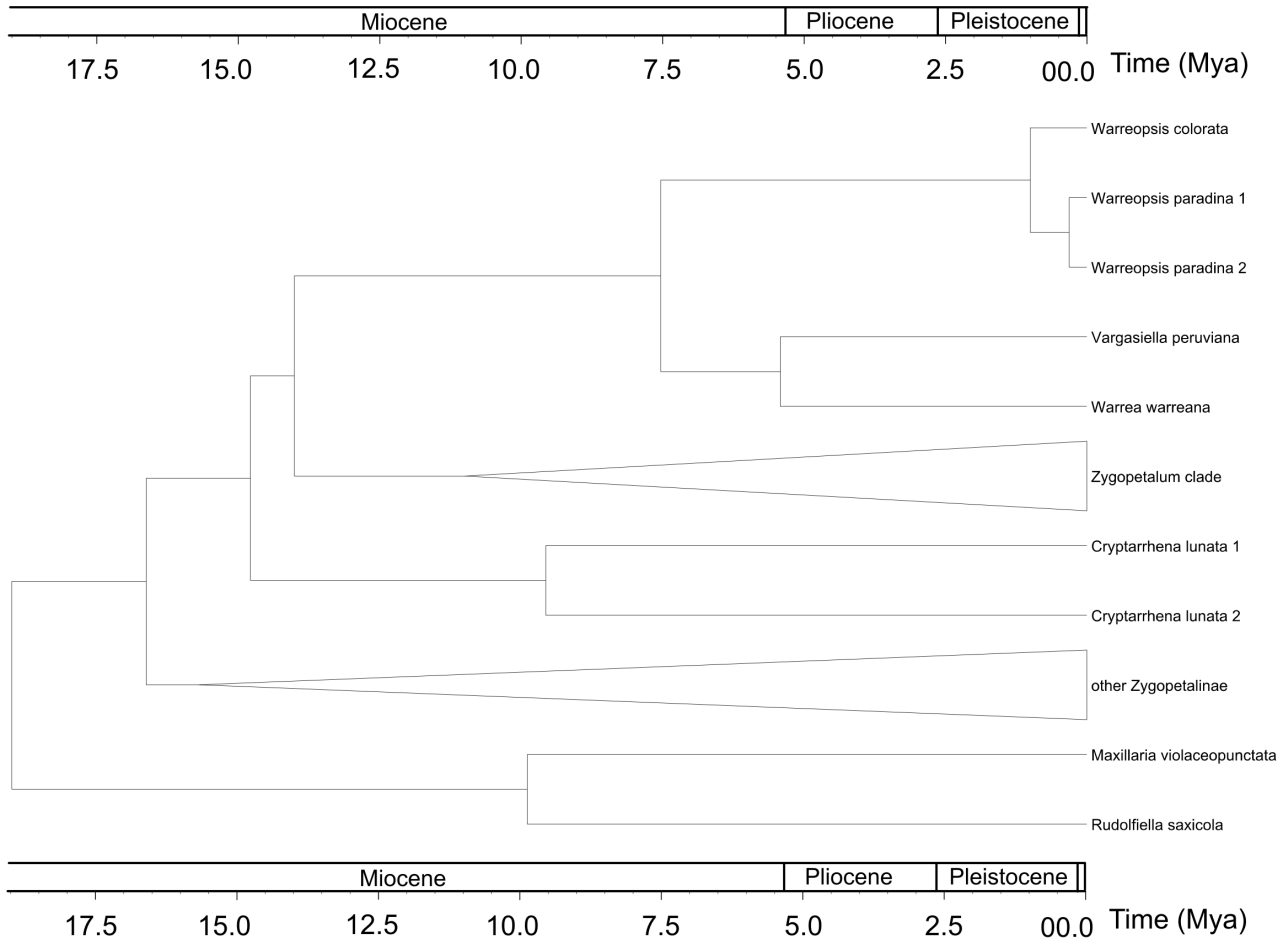
Clocktree of Zygopetalinae estimated from the *matK* gene.

The topology of the tree within Zygopetalinae estimated from the *mat*K gene are generally congruent with those from the parsimony analysis (Fig. 4). The most recent common ancestor of *Vargasiella, Warrea* and *Warreopsis* originated in the Miocene around 7.5 Ma ago. The divergence time for *Vargasiella* and *Warrea* was estimated for approximately 5.4 Ma ago (Miocene/Pliocene).

### Macromorphological characters

**Figure 5:**
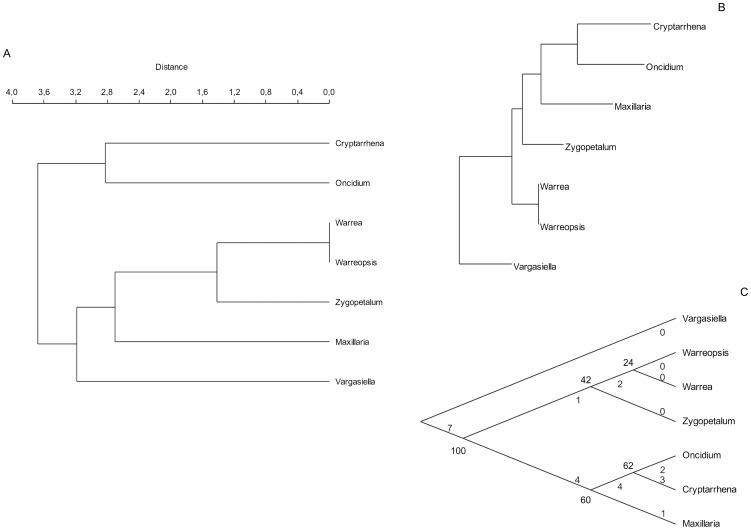
Results of phenetic analysis. (A) UPGMA dendrogram, Euclidean distance (B) Neighbor joining dendrogram, Euclidean distance (C) Parsimony heuristic search TBR cladogram, Fitch optimization with bootstrap.

Regardless of the method of clustering *Vargasiella* is well separated from all other taxa used in used in phenetic and cladistic analyses (Fig. 5). The genus either occupies the basal position in Zygopetalinae s.l. comprising a branch (UPGMA based phenogram) or the basal position of the whole tree (Neighbor joining and Maximum parsimony), which clearly indicates that there is a morphological gap between *Vargasiella* and other genera. In Table 2 we present the most important morphological characters of *Vargasiella, Warrea* and *Warreopsis*. Admittedly, both taxa could be regarded as related as far as molecular analyses are concerned;, however, based on morphological characters, their relation can be questioned.

**Table 2:** Comparison of main morphological characters between *Vargasiella*, *Warrea*, *Warreopsis, Maxillaria* and *Oncidium*.

Character	*Vargasiella*	*Warrea*	*Warreopsis*	*Maxillaria*	*Oncidium*
Pseudobulbs	absent	homoblastic	homoblastic	heteroblastic	heteroblastic
Leaves	convolute	convolute	convolute	conduplicate	conduplicate
Inflorescence	subapical,	subbasal,	subbasal,	basal, single-	basal, many-
	many-	many-	many-	flowered	flowered
	flowered	flowered	flowered		
Lip	simple,	simple,	simple,	more or less	sessile,
	oblong to	flabellate	flabellate,	prominently	variously
	elliptic,	sessile	shortly	3-lobed,	lobed
	sessile to		clawed	sessile	
	clawed				
Callus	obscure	radiating	prominent,	simple,	often a
		from the base	flabellate	oblong	complex
		along nerves			structure
Gynostemium	erect, rather	elongate,	elongate,	elongate or	slightly arched
	short	slightly	slightly	short, slender	or erect, rather
		arched,	arched,	or stout,	stout
		robust	robust	usually	
				gently	
				arched,	
				occasionally	
				suberect	
Column foot	short	short, but	obscure	usually half	absent
		robust, gently		as long as	
		narrowing		column part,	
		towards the		occasionally	
		apex		longer than	
				column part	
Stigma	large, deltoid,	rather large,	rather large,	large, elliptic,	large, ovate to
	deeply	elliptic,	elliptic,	deeply	elliptic, deeply
	concave	deeply	deeply	concave	concave
		concave,	concave,		
		partially	partially		
		hidden by	hidden by		
		rostellum	rostellum		
Anther	ventral,	ventral,	ventral,	subapical,	subapical,
	transversely	incumbent,	incumbent,	incumbent,	incumbent,
	ellipsoid,	ellipsoid-	ellipsoid-	ellipsoid-	ovoid
	dorsiventrally	ovoid,	ovoid,	ovoid or	
	flattened	dorsiventrally	dorsiventrally	obovoid	
		compressed	compressed		
Pollinia	4 in two	4, in two	4, in two	4, in two	2, subglobose,
	pairs,	pairs, almost	pairs, almost	pairs,	slightly
	subequal,	superposed,	superposed,	unequal in	dorsiventrally
	obovoid-	unequal in	unequal in	size,	flattened,
	ellipsoid	size,	size,	dorsiventrally	hard,
		dorsiventrally	dorsiventrally	compressed,	unequally and
		compressed,	compressed,	almost flat or	deeply cleft,
		ellipsoid-	ellipsoid-	concave on	empty inside
		ovoid, rather	ovoid, rather	ventral	
		hard	hard	surface and	
				slightly	
				convex on	
				the outer one,	
				obovoid to	
				ellipsoid,	
				rather soft	
Tegula	single,	single,	single,	single,	single, oblong,
	oblong,	oblong,	oblong,	elliptic-ovate	thin, lamellate
	lamellate	widest near	attenuate	to crepiform,	
		the base,	towards acute	thin,	
		narrowed	apex,	lamellate	
		gradually	lamellar		
		towards the			
		apex, thin,			
		lamellar			
Viscidium	elliptic-	single,	single,	single, very	single, oblong
	cordate,	elliptic-ovate	elliptic-ovate,	narrow,	elliptic, thick,
	distinctly 2-	or elliptic-	lamellar	transversely	fleshy
	lobed at the	rhombic,		elliptic,	
	apex	attenuate		triangular or	
		towards both		crepiform,	
		apex and		thin lamellate	
		base, lamellar			
		thin			
Rostellum	short and	rather large,	relatively	dome-like,	short, conical-
	broad	dome-like, 3-	short,	broad and	digitate in the
		lobed, the	dome-like, 3-	short,	middle, obtuse
		middle lobe	lobed, the	shallowly	
		ligulate-	middle lobe	sinuous in	
		triangular,	ligulate, side	front	
		acute, side	lobes obscure		
		lobes			
		obscure,			
		separated by			
		shallow sinus			
		from the			
		middle lobe			
Rostellum remnant	with short	3-lobed, the	3-lobed	deeply	bilobulate at
	apiculus in	middle lobe		notched at	the middle,
	the middle	subulate,		the apex	with oblique
		acute, rigid,			shallowly
		both side			concave plate
		lobes much			between acute
		reduced,			lobules
		obtuse			

### Micromorphological features

**Figure 6:**
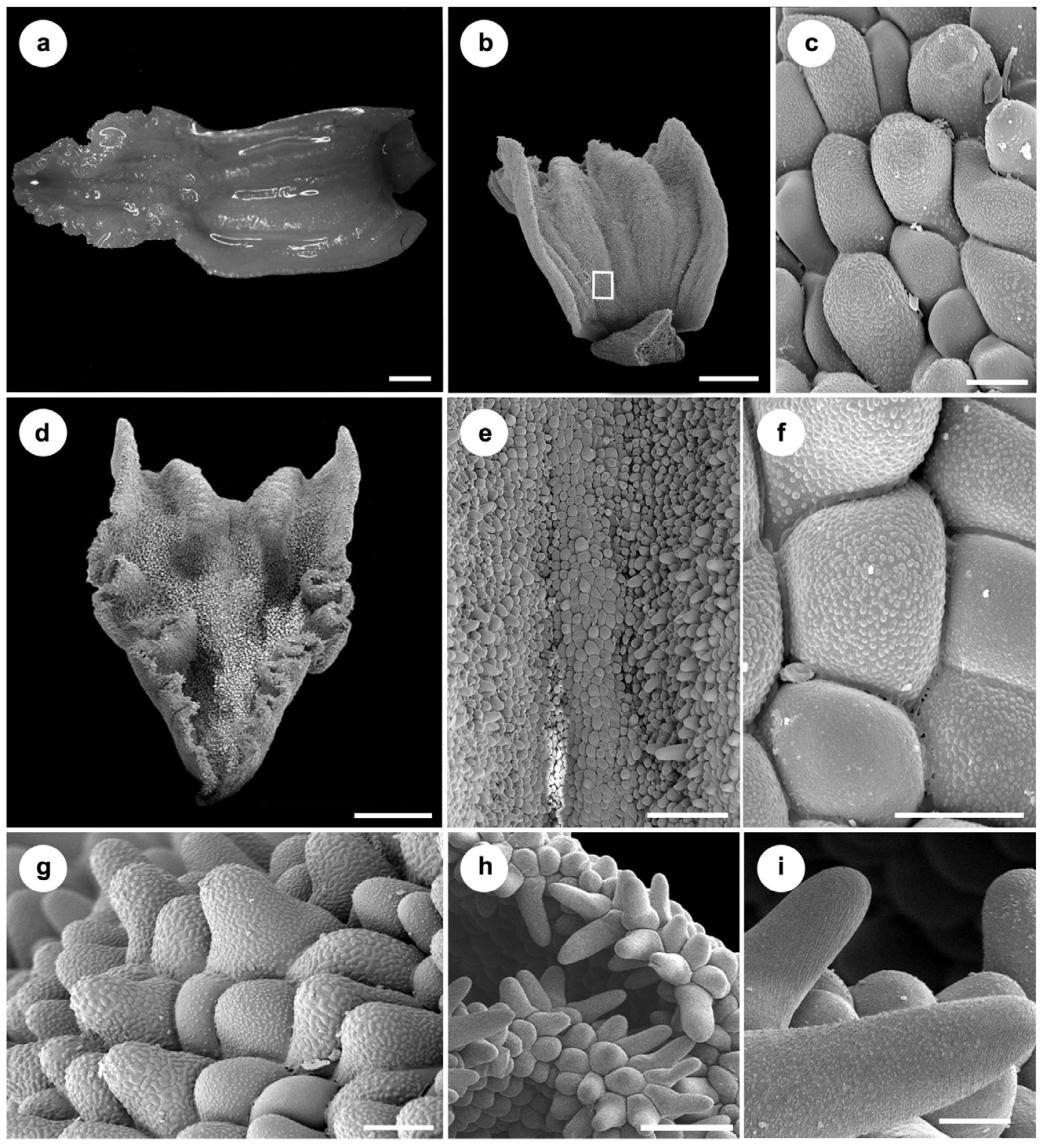
*Vargasiella venezuelana* C.Schweinf. (A) Lip with central fleshy disc divided into two thickenings. (B) Lip base. (C) Details of (B): the irregularly sized rounded cells to obpyriform and conical papillae, visible cuticle swellings on the cells. (D) Lip middle part and apex. (E) Detail of (D). Surface between thickenings built by the obpyriform to slightly conical papillae (F). (G) Conical papillae present on the thickenings; Visible cuticle swellings on the cells (F-G). (H) The involute undulate margins built by groups of elongated conical papillae. (I) Papillae (detail of H) covered by undulated cuticle, cuticle swellings also present.

**Figure 7:**
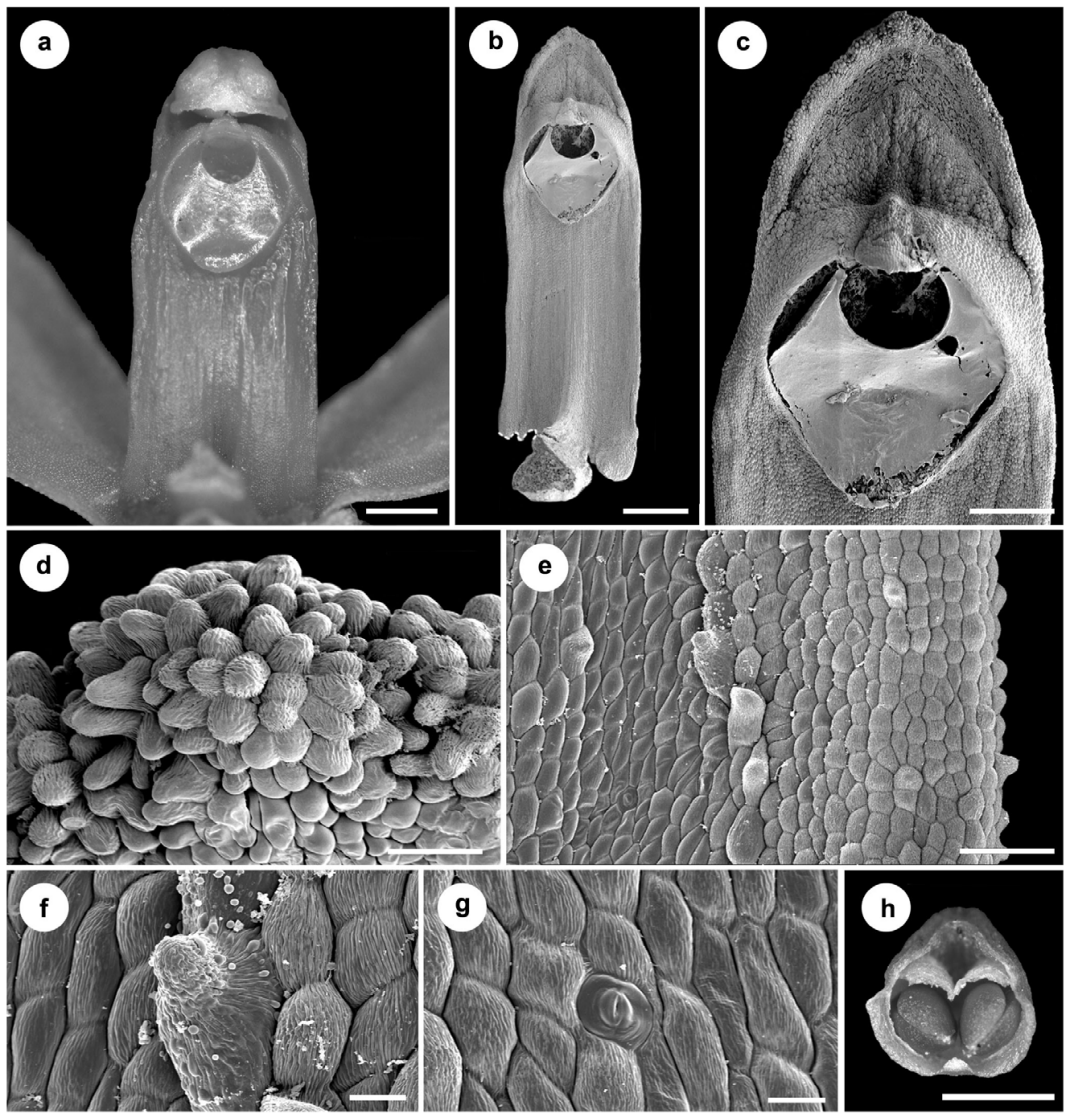
*Vargasiella venezuelana* C.Schweinf. (A-C) The stout short gynostemium with large, deltoid, deeply concave stigma (A: LM; B-C: SEM). (D). Rounded to conical papillae occurring on the edge of clinandrium covered by ridged cuticle. (E) Surface under the stigma covered by flat cells with regularly ridged cuticle (also visible in F-G); a few longer conical papillae noticed close to the column margins (details in F) with visible drops of secretory remnants on surface. (G) Paracytic type of stomata. (H) The four obovoid-ellipsoid pollinia grouped in two unequal pairs.

The characteristic feature of the lip of *Vargasiella peruviana* is the presence of the central fleshy disc which is divided into two calli (Fig. 6A) starting from the base (Fig. 6A-B, D). The epidermis of the lip base is built by irregularly sized rounded cells to obpyriform and conical papillae (Fig. 6C). Between the thickenings (Fig. 6D-E), obpyriform to slightly conical papillae are noticed (Fig. 6E-F). The bigger conical papillae are distinctly visible on the calli (Fig. 6G). The cuticle swellings are noticed on the whole inner lip surface (Fig. 6C, F, G, I). The involute an undulate margins (Fig. 6H) consist of groups of elongated conical papillae, covered by undulated cuticle (Fig. 6I). The stout, short gynostemium with a large, deltoid, deeply concave stigma (Fig. 7A-C) is covered by flat cells with a regularly ridged cuticle (Fig. 7E-G) or conical papillae (Fig. 7D-F). The rounded to conical papillae occurring on the edge of the clinandrium (Fig. 7C-D) are covered by a cuticle with thick ridges. Under the stigma, close to the column margins, a few longer papillae are noticed (Fig. 6E) with visible drops of secretory remnants on their surface (Fig. 7F). The paracytic type of stomata is present (Fig. 7G). The four obovoid-ellipsoid pollinia are grouped in two pairs (Fig. 7H).

**Figure 8:**
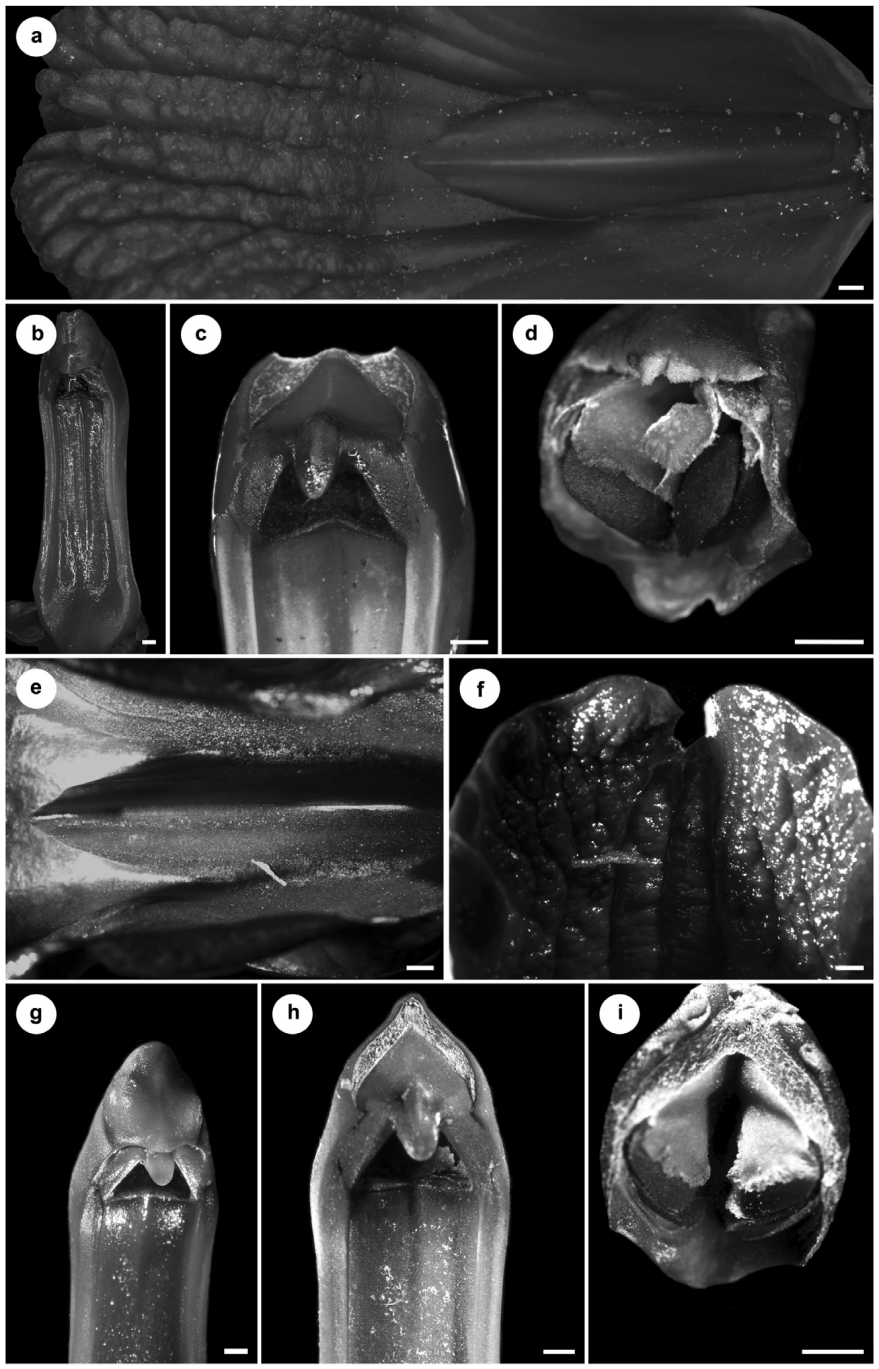
*Warrea costaricensis* Schltr. (A) Lip with central single callus beginning from the smooth base, deeply undulate surface at the lip apex. (B) The elongate, robust gynostemium. (C) Details of (B): tegula thin, narrowed gradually towards apex, stigma rather large, elliptic, deeply concave, partially hidden by rostellum. (D) Polinia four in two unequal pairs. *Warrea warreana* (Lodd. *ex* Lindl.) C. Schweinf. (E) Lip base with central callus. (F) Deeply undulate lip apex. (G-H) Gynostemium with thin tegula and elliptic stigma (the same as in *W. costaricensis*). (I) Polinia four in two unequal pairs.

**Figure 9:**
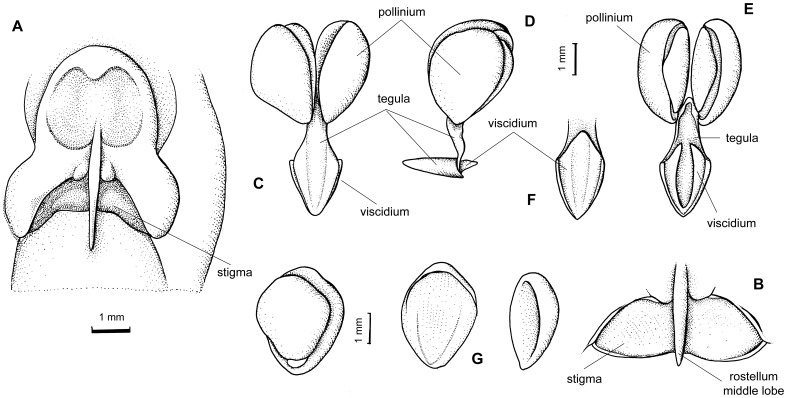
*Warrea hookeriana* (Rchb.f.) Rolfe. (A,B) Rostellum remnant, front view. (C-E) Various views of pollinarium. (F) Viscidium, bottom view. (G) Pollinia, various views (cult. Glasnevin BG, K).

Several differences were observed in the morphology of the lip and gynostemium between *V. peruviana* (Fig. 7A-H), *Warrea costaricensis* Schltr. (Fig. 8A-D) and *W. warreana* (Lodd. *ex* Lindl.) C. Schweinf. (Fig. 8E-F). In both *Warrea* species the central, single callus starts from the smooth lip base (Fig. 8A, E), but the surface of the lip apex is deeply undulate (Fig. 8A, F). The gynostemium is erect, rather short, clavate (Fig. 8B, G, Fig. 9), with a short column foot. The tegula is thin, narrowing gradually towards the apex (Fig. 8B-C, G-H, Fig. 9C, D, E). The rather large, elliptic and deeply concave stigma is partially hidden by the rostellum (Fig. 8B-C, G-H). The four ellipsoid-ovoid pollinia are arranged in two unequal pairs (Fig. 8D, I, Fig. 9G).

### Ecological niche modeling evaluation and the distribution factors

All repeated ecological niche models received AUC values above 0.9 (Table S2), which confirms the very high sensitivity of the analysis - the models accurately predict the value of an observational response [35]. The high AUC scores are partially related to the small sample size, especially in *Warreopsis* models; however; the low standard deviation for all studied taxa observed between the replicate runs for all the studied taxa indicates that all the created models are clustered closely to the mean.

From the studied bioclimatic variables the isothermality (bio3) and altitude (alt) were the most influential bioclimatical factors for *Vargasiella* distribution. Some contribution to the models derived also from the temperature seasonality.

The variables limiting the distribution of *Warrea* and *Warreopsis* species are similar; however, a larger impact is observed in the case of precipitation (upon the impact of the elevation recognized in *Vargasiella*). The potential range of *Warrea warreana* and *Warreopsis pardina* is related to the amount of rainfall in the warmest quarter of the year (bio18), while the total annual precipitation (bio12) defines the geographical limits of *Warrea costaricensis* and *Warreopsis parviflora*. A comparison of the percentage contribution of the three most important variables to the models created for the studied taxa is given in Table 3.

**Table 3:** Contribution of most important bioclimatic variables to the ecological niche models of the studied taxa.

Taxon	Model	Var_1 (% contribution)	Var_3 (% contribution)	Var_3 (% contribution)
*Vargasiella* sp.	LGM model	Bio3 (56.5)	alt (22.8)	Bio4 (8.4)
*Vargasiella* sp.	Present-time model	Bio3 (53.4)	alt (22.5)	Bio4 (8.5)
*Warrea costaricensis*	LGM model	Bio12 (26.1)	Bio4 (22.9)	Bio3 (14.7)
*Warrea costaricensis*	Present-time model	Bio12 (31.9)	Bio4 (18.9)	Bio3 (12.8)
*Warrea warreana*	LGM model	Bio3 (44.2)	Bio18 (13.9)	Bio4 (13.1)
*Warrea warreana*	Present-time model	Bio3 (45)	Bio18 (16.2)	Bio4 (12)
*Warreopsis pardina*	LGM model	Bio3 (49.6)	Bio4 (17.1)	Bio18 (12.9)
*Warreopsis pardina*	Present-time model	Bio3 (54.1)	Bio4 (15.4)	Bio18 (9.4)
*Warreopsis parviflora*	LGM model	Bio3 (24.6)	Bio12 (18.8)	Bio4 (18)
*Warreopsis parviflora*	Present-time model	Bio3 (23.6)	Bio4 (21.9)	Bio12 (17.8)

### Postulated LGM refuge areas

**Figure 10:**
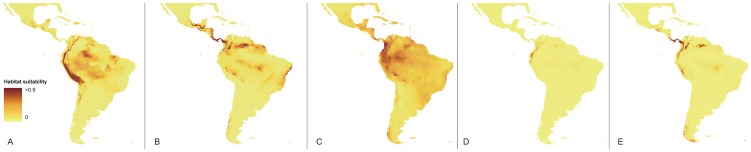
Distribution of the suitable habitats of *Vargasiella* (A), *Warrea costaricensis* (B), *Warrea warreana* (C), *Warreopsis pardina* (D) and *Warreopsis parviflora* (E) during LGM.

The most suitable habitats for *Vargasiella* during the last glacial maximum (LGM) were probably distributed in the montane regions from the southern part of the Colombian Central Cordillera to the Bolivian Cordillera Real (Fig. 10A). Some potentially available but less suitable niches were probably also located around Pico da Neblina on the border between Venezuela and Brazil as well as near the Brazilian Mato Grosso do Sul. All the identified areas correspond, during LGM, to rock deserts and semi-deserts characterized by low vegetation cover [36].

On the other hand the most suitable habitats for both *Warrea* species were located in the northern part of the South American Pacific coast (*W. warreana*, Fig. 9C) and along the Panamanian Isthmus (*W. costaricensis,* Fig. 10B). The designated refuge areas were covered in LGM with the tropical rainforest that corresponds to Olsons' seasonal tropical forest and broad-leaved humid forest (including swamp forest).

The estimated glacial refugia of *Warreopsis* species differ significantly between the studied species. The suitable ecological niches of *W. pardina* were limited to the lower parts of the Andean slopes from Peru to Colombia (Fig. 10D), which correspond to Olsons' savannas and woodlands [36] characterized by the presence of leaf cover above 80 cm from the ground. The suitable habitats for *W. parviflora* were more widespread and they were located both in the lower montane regions (Andes, Santa Marta, Talamanca) and highlands (Guiana Highlands) as well as in lowland areas (Darién Gap, Fig. 10E). The estimated refugia were covered by Olsons' [36] woodlands, seasonal tropical forest and swamp forest.

Based on the niche overlap statistics the habitats occupied by *Vargasiella* species varied significantly from all other studied taxa. The most similar niches were occupied by *Warrea warreana* (D = 0.4830, I = 0.7632) while the greatest differences are observed in comparison with *Warreopsis parviflora* (D = 0.2148, I = 0.4565). The glacial habitats of both *Warrea* species were similar (D = 0.4753, I = 0.7436) whereas the suitable niches of *Warreopsis* varied slightly between the studied species (D = 0.3404, I = 0.6065).

All calculations of the similarities between the geographical distribution of the niches occupied in LGM by all studied taxa are given in Tables 4–5.

**Table 4:** The glacial niche overlap statistics – Schoener's D statistic.

	*W. warreana*	*W. costaricensis*	*Vargasiella* sp.	*Warreopsis pardina*	*Warreopsis parviflora*
*W. warreana*	x	0.4753	0.4830	0.2713	0.2770
*W. costaricensis*	0.4753	x	0.2901	0.2779	0.4737
*Vargasiella* sp.	0.4830	0.2901	x	0.3125	0.2148
*Warreopsis pardina*	0.2713	0.2779	0.3125	x	0.3404
*Warreopsis parviflora*	0.2770	0.4737	0.2148	0.3404	x

**Table 5:** The glacial niche overlap statistic - I statistic.

	*W. warreana*	*W. costaricensis*	*Vargasiella* sp.	*Warreopsis pardina*	*Warreopsis parviflora*
*W. warreana*	x	0.7436	0.7632	0.5138	0.5610
*W. costaricensis*	0.7436	x	0.5429	0.5377	0.7677
*Vargasiella* sp.	0.7632	0.5429	x	0.5800	0.4565
*Warreopsis pardina*	0.5138	0.5377	0.5800	x	0.6065
*Warreopsis parviflora*	0.5610	0.7677	0.4565	0.6065	x

### Current potential range

**Figure 11:**
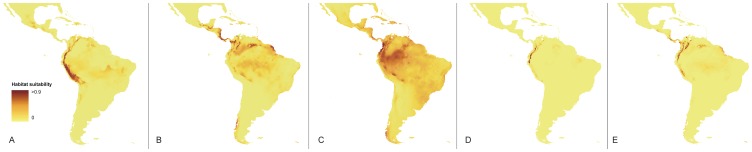
Current distribution of the suitable habitats of Vargasiella (A), Warrea costaricensis (B), Warrea warreana (C), Warreopsis pardina (D) and Warreopsis parviflora (E).

Compared to the LGM range, the current distribution of the habitats suitable for the occurrence of the *Vargasiella* species is significantly limited both longitudinally as well as latitudinally. The available niches are concentrated from the Ecuadorian and Peruvian Andes to the Altiplano in the south. There is no additional suitable location outside the Andean region (Fig. 11A).

The current potential ranges of both studied *Warrea* species are discontinuous. The suitable niches of *W. costaricensis* are distributed along lower parts of Cordillera de Talamanca to Cordillera de Salamanca (Fig. 11B). The other available habitats are located in the Darién region and the northern foothills of the Western Andean Cordillera as well as in eastern slopes of the Eastern Cordillera. The most suitable habitats for *W. warreana* are located on the Pacific coast of northern South America; however, the essential part of the Amazon basin is characterized by somewhat less favorable, but possibly tolerable bioclimatic conditions (Fig. 11C).

The suitable habitats for *Warreopsis pardina* are restricted to the lower parts of the northern Andean range from Colombia to the Chamaya river in the south, but the species has not been reported from Peru so far (Fig. 11D). The niches of *Warreopsis parviflora* are distributed irregularly along the northern Andes and Cordillera de Talamanca. Additional potentially available habitats are located in the Santa Marta mountains (Colombia) and Guiana Highlands (Fig. 11E). It is noteworthy that so far this species has only been collected in Central America.

The similarities between the geographical distribution of the suitable niches of all studied taxa are given in Tables 6–7.

**Table 6:** The niche overlap statistics – Schoener's D statistic.

	*Vargasiella* sp.	*Warrea warreana*	*Warrea costaricensis*	*Warreopsis pardina*	*Warreopsis parviflora*
*Vargasiella* sp.	x	0.3952	0.3812	0.2590	0.2576
*Warrea warreana*	0.3952	x	0.5650	0.2085	0.3272
*Warrea costaricensis*	0.3812	0.5650	x	0.2300	0.5434
*Warreopsis pardina*	0.2590	0.2085	0.2300	x	0.3859
*Warreopsis parviflora*	0.2576	0.3272	0.5434	0.3859	x

**Table 7:** The niche overlap statistics - I statistic.

	*Vargasiella* sp.	*Warrea warreana*	*Warrea costaricensis*	*Warreopsis pardina*	*Warreopsis parviflora*
*Vargasiella* sp.	x	0.6754	0.6459	0.5219	0.4840
*Warrea warreana*	0.6754	x	0.8001	0.4337	0.5863
*Warrea costaricensis*	0.6459	0.8001	x	0.4779	0.8145
*Warreopsis pardina*	0.5219	0.4337	0.4779	x	0.6610
*Warreopsis parviflora*	0.4840	0.5863	0.8145	0.6610	x

### Range and niche shift

While the current and LGM estimated distribution of the suitable habitats seem to be similar, the calculated values indicate the significant differences in both, ranges and niches occupied by *Vargasiella* in the studied time periods. The whole potential range overlap value is just 0.361 and the niche overlap statistics are: D = 0.279, I = 0.572. The same calculations made for models of the studied *Warrea* and *Warreopsis* species revealed a rather insignificant shift in the occupied areas and niches between LGM and the present time (Table 8).

**Table 8:** Overlapping of the ranges and occupied niches between LGM and present time.

Taxon	Range overlap test	D	I
*Vargasiella* sp.	0.361	0.279	0.572
*W. costaricensis*	0.761	0.716	0.924
*W. warreana*	0.934	0.930	0.994
*W. pardina*	0.748	0.708	0.922
*W. parviflora*	0.620	0.592	0.831

## Discussion

The taxonomic position of *Warrea* and *Warreopsis* is rather obvious. Almost all orchid taxonomists classify them usually within the variously defined Zygopetalinae ([2] [3] [7] [9]). However, Szlachetko [8] separated *Warrea*, *Otostylis* Schltr., *Warreella* Schltr. and *Warreopsis* Garay from Zygopetalinae and united them in the subtribe Warreinae. This subtribe has been proposed as a taxon clustering those genera different from all other Zygopetaleae *sensu* Szlachetko [8] by having slender, homoblastic pseudobulbs and convolute, plicate leaves. All the genera share a similar gynostemium structure, i.e., usually a short column foot, 4 superposed, unequal pollinia, a long rostellum, lamellar tegula and viscidium forming together a kind of sheath around the rostellum core.

*Warrea/Warreopsis* and *Vargasiella* differ one from another in their stem architecture, and leaf and lip structure. Even though they are characterized by the presence of four superposed pollinia arranged in two pairs, the rostellum structure is essentially different. In *Warrea, Warreopsis* and other genera of Zygopetaleae *sensu* Szlachetko [8] the rostellum is 3-lobed, with the middle lobe being ligulate-triangular, acute, springy and obscure lateral lobes, separated from the middle one by a shallow sinus. The tegula and viscidium are thin, lamellar, more or less elongate and together they form a sheath around the rostellum core. The stigma is very narrow, hidden in the most part by the rostellum. In contrast, the rostellum of *Vargasiella* is short and broad and its major part becomes transformed into the viscidium and tegula, and when removed with the pollinia drops out as accessories. The tegula is small and the viscidium even smaller, and distinctly bilobed. The receptive surface is broad, easily accessible to pollen mass. Additionally, the genera in question are distinguishable by the form of the rostellum remnant. In *Warrea*, the rostellum after removal of the pollinarium is 3-lobed, the middle lobe is subulate, acute, rigid, both lateral lobes are much reduced, obtuse. In the closely related *Warreopsis* the rostellum remnant is similar, with the middle lobe being shorter. In contrast in *Vargasiella*, the rostellum remnant consists of a short, fleshy apiculus in the middle. The question is why we consider the rostellum structure in detail? The rostellum evolved from an altered middle lobe of the stigma and it plays a crucial role in the pollination of orchid flowers. First, it produces important accessories for pollinia, i.e., the viscidium and tegula, the structures that enabled their transfer by a pollinator. Secondly, in many species the rostellum forms a kind of barrier between the anther and the receptive surface suppressing autogamy. The rostellum of *Warrea* and *Warreopsis* are such cases. The short and erect rostellum of *Vargasiella* is not a sufficient barrier to prevent autogamy. A similar type of gynostemium is found in *Maxillaria*. The rostellum is short and broad, dome-like and the stigma is relatively large, not hidden by the rostellum. Both rostellar products are similar to those of *Vargasiella*, i.e. the tegula is rather small and the viscidium distinctly bilobed, hippocrepiform and does not form a sheath.

**Figure 12:**
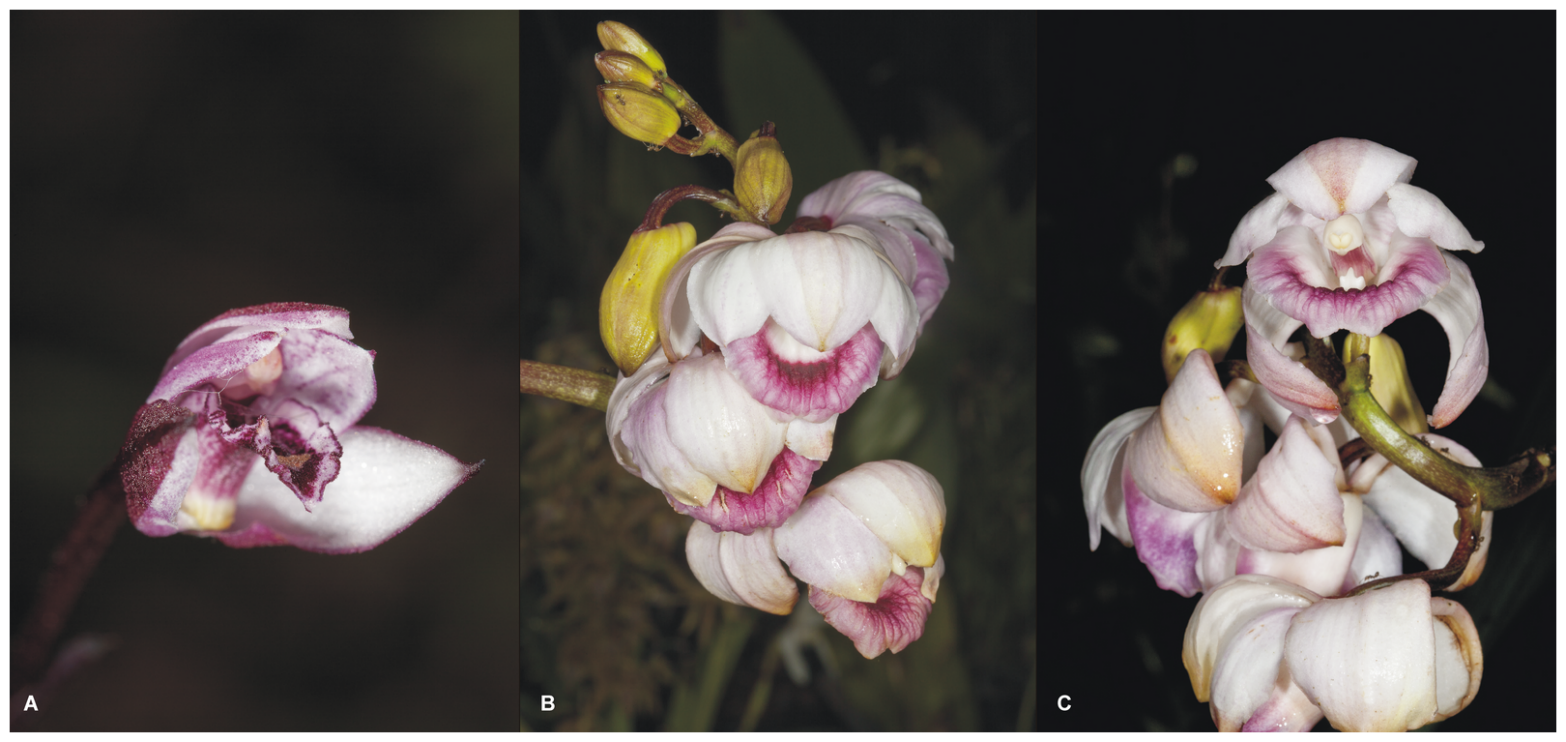
Flowers of *Vargasiella peruviana* (A) and *Warrea warreana* (B-C). Photos: T. Kusibab.

The pollinators' observations would be useful to understand the function of these floral structures. Unfortunately, nothing is known about pollination biology of *Vargasiella*, or that of *Warrea* or *Warreopsis*. Pupulin [37] considered that *Warrea* is probably pollinated by male euglossine bees. Based on the flower colour and its morphology (Fig. 12) and the knowledge that most Zygopetalinae are pollinated by euglossinae bees [38], we can predict that *Vargasiella* are exploits the same pollinators. The cuticle swellings visible on the lip surface may be the fragrance which is collected by male euglossinae bees, but this hypothesis needs more detailed studies.

The number of known populations of *Vargasiella* is very small, and the information about its ecological interactions and habitat requirements is equally limited. The quick organic matter decomposition in the tropical regions together with the anatomical structure of the studied species (i.a. the thin coat of the microscopic seeds, and thin cuticule) have made them undetectable in fossil material and this precludes conducting traditional paleobotanic research including palynological procedures.

The presented results of the ENM analysis based on presence-only data clearly indicate the Central Andes as the most probable glacial refugium of *Vargasiella*. The statistical calculations of the ranges and niches overlap between models for the studied time periods indicate not only the current regression of *Vargasiella* range, but also the niche shifting in response to warming climate after the LGM. This kind of range reduction is primarily the result of distributional shifts and fragmentation of primary habitats as well as the expansion of unsuitable climatic conditions exceeding species' ecological tolerance [39] [40]. The exact opposite situation is observed with respect to the other two studied genera. The estimated suitable habitats for the occurrence of *Warrea* and *Warreopsis* species during LGM were probably located in warmer lowland to lower montane regions of Mesoamerica and Northern South America and no significant changes in their geographic ranges or occupied niches are observed in the present time. The finding is important especially in terms of plant speciation. Wright [41] suggested that ecological shifts are the most likely basis for the origin of genera and these shifts seem to be punctuational rather than gradual [42] [43].

We decided to include the results of niche modeling in this study, despite the fact that we are aware that the results of ENM analysis may overestimate the distribution of the suitable niches of the studied taxa, mainly due to the small sample size. The models created for the present time are rather consistent with the occurrence data of the species, even though not all of them were included in the analysis because it was not possible to georeference their locations. We believe that in the case of poorly known taxa, such as *Vargasiella*, which are untraceable in fossil material, the use of the algorithms for computing the distribution of their suitable niches in the past is the only method to recognize their areas of historical stability and hereby to analyse the changes in their ecological niches.

*Vargasiella* species can be defined as psychrophytes usually growing in wet and fairly cool conditions, in submontane and montane forest and forest edges, about 2100–3400 m a.s.l. The occurrence of *V. venezuelana* has been reported from *Bonnetia* forest (*Steyermark 74914*, F), while *V. peruviana* has also been reported from stunted cloud forest (*Foster & Smith 9094*, MO; *Gentry & Smith 35984*, MO). *Warrea* species occupy dense, rain and moist forest, growing in decaying leaf litter in the shade, usually below 1500 m a.s.l, and often near rivers and streams (*Binacional 1104*, MO; *Dressler 3250,* MO). *Warrea warreana* has been reported growing epiphytically on tree ferns (*Zuloaga & al. 6898*, SI). Studied *Warreopsis* species are found in lower montane and montane areas growing terrestrially (rarely epiphytically) in wet and very wet forests (*Haber & al. 4510, 4484, MO*; *Davidse & Pohl 1692A*, MO).

The ENM results indicate that the occurrence of *Vargasiella* and *Warrea* species is limited by similar bioclimatic variables, mainly by isothermality. However, the distribution of *Vargasiella* is seriously influenced by the elevation above sea level and this is probably the crucial environmental factor resulting in the separation of suitable habitats and thereby the ranges of the studied taxa. The habitat requirements of *Vargasiella clearly* differ from those of *Warrea* and *Warreopsis*, as indicated by the scores of the niche overlap test.

The clocktree of Zygopetalinae indicated that the most recent common ancestor of *Vargasiella, Warrea* and *Warreopsis* could probably originate from around 7.5 Ma ago, while the divergence time for *Vargasiella* and *Warrea* was estimated for approximately 5.4 Ma ago. These events may be easily linked to the major geological changes in South America. The rapid rise of the Andean plateau took place in the late Miocene (10.4–5 Ma ago) and this was related with the establishment of dramatic precipitation gradients perpendicular to the orogen, and changes in tectonic processes in the Andean orogenic wedge [44]. These changes also influenced the Amazon region and during the Pliocene the lowland fluvial systems of southwestern Amazonia become isolated from the Andes by the newly formed fluvial systems (Ucayali and Madre de Dios). In the early Pliocene the Amazon fluvial system integrated regionally and acquired its present appearance [45].

## Conclusions

In the genetic analyses *Vargasiella* is placed within a highly supported subclade together with *Warrea* and *Warreopsis* that is situated at the base of the Zygopetalinae *sensu lato* clade. However, the relationships between *Warrea, Warreopsis* and *Vargasiella* are not resolved.

Significant differences in the presence of storage organs, leaf structure, inflorescence type and lip form were observed in the macromorphological studies of *Vargasiella* and related taxa. Moreover, differences in the micromorphology of the lip and gynostemium between *V. peruviana, Warrea costaricensis* and *W. warreana* were found.

The ecological niche modeling suggests regression of *Vargasiella* range since LGM. Moreover, unlike in *Warrea* and *Warreopsis* a niche shift in response to postglacial climate changes was observed in *Vargasiella*.

The genus *Vargasiella* appears to be an outshoot of the main branch of evolution of Zygopetaleae. Interestingly, the *Vargasiella*-*Warrea* dichotomy could have taken place ca 5.4 Ma ago, later than the divergence of *Warreopsis* from the mutual lineage (ca 7.5 Ma ago, Fig. 4). Considering the morphological data and the results of molecular analyses we therefore formulate a hypothesis that *Vargasiella* and *Warrea* may have evolved from a common ancestor. Accumulation of morphological differences and acceleration of the evolution of *Vargasiella* were faster than in other Warreinae, a feature which could probably be synchronized with adaptation to cooler and wetter conditions. This can be supported by the observations made on *Warrea* and *Warreopsis*, both inhabiting similar ecological niches. Both are more reminiscent of one another considering morphological characters than could be speculated based on molecular analysis outcomes.

## Taxonomic Treatment

***Vargasiella*** C.Schweinf., Bot. Mus. Leafl., Harvard Univ. 15: 150. 1952. Type: *Vargasiella peruviana* C.Schweinf.

The genus includes two species, they can be distinguished as follows:

1. Sepals and petals subsimilar, lip sessile, base truncate-subcordate, ovate-lanceolate, with calli …………….***V. peruviana***

1. Sepals and petals dissimilar, lip clawed, base cordate, elliptic-suborbicular, ecallose ………. ***V. venezuelana***

***Vargasiella peruviana*** C.Schweinf., Bot. Mus. Leafl., Harvard Univ. 15: 150, tab. 47. 1952; Type: Peru, Convención, hills of Pintobamba, in humus forest, perianth white with pinkish lip. *Vargas 3288* (holotype AMES! - type illustration). Cuzco: Paucartambo, Pillahuata, floral segments white lined with pink. *Vargas 3010* (paratype AMES!).

Plant epiphytic or terrestrial, slender. Stem elongate, decumbent, producing scattered fibrous, short, densely tomentose and stout roots in the lower part, leafy in the upper part, entirely concealed by tubular sheaths. Leaves 5.6–13.5 cm long, 2–2.5 cm wide, several, distichous, convolute, elliptic to oblong-elliptic or elliptic-lanceolate, acuminate, cuneate below, sessile or indistinctly petiolated, articulated to close tubular sheaths, membranaceous, 3- to 5-nerved. Inflorescence up to 33.5 cm long, arising from the axil of an upper leaf, erect, racemose; peduncle 21.5 cm long with several remote, tubular below and lanceolate above sheaths; raceme loosely up to 15-flowered. Flowers medium-sized, subfleshy, perianth white with pinkish lip. Floral bracts up to 14 mm long, oblong, acute, spreading. Ovary prominently 6-sulcate. Dorsal sepal 13.2 mm long, 6 mm wide, ovate-oblong, acute to mucronate, concave, 5-nerved, with margins very minutely cellular-erose. Lateral sepals 14.5 mm long, 7 mm wide, similar, ovate-oblong, acute, cymbiform, dorsally carinate with the keel produced into a conspicuous mucronate apex, 5- or 6-nerved, lightly oblique. Petals 12 mm long, 6 mm wide, elliptic-ovate, acute to apiculate, 5-nerved. Lip 10 mm long, 6 mm wide, simple, arcuate-recurved and parallel to the column with the sides erect in natural position, articulated to the column foot, with the anterior margins strongly undulate, disc when expanded ovate-oblong, cordate at base, rounded and acute or apiculate at apex, furnished with two fleshy thickenings in the lower half. Gynostemium 7 mm long, stout, with a narrow fleshy wing on each side throughout, subtruncate above. Anther relatively small, 1-celled, galeate. Pollinia 4, in two unequal pairs, without appendages, strongly complanate-subglobose, waxy.

Flowering time: Throughout the year.

Distribution: Peru, Bolivia. Elev. 2400–3400 m a.s.l.

***Vargasiella venezuelana*** C.Schweinf., Bot. Mus. Leafl., Harvard Univ. 18: 219, tab. 44. 1958; Type: Venezuela, Bolívar. Chimantá Massif, northwestern part of summit of Abácapa-tepuí, in Bonnetia forest. *Steyermark 74914* (holotype AMES! - type illustration, isotype F!).

Plant terrestrial, robust, up to 187.5 cm long with decumbent stem, sparsely rooting. Roots solitary, very few, fibrous, rather stout, sparsely pubescent. Stem entirely concealed by appressed, imbricating, evanescent, leaf-bearing, tubular sheaths; the lower ones scarious and disintegrating into fibers, the middle and the upper ones green and leaf-bearing. Leaves 14-17 cm long, up to 3.3 cm wide, elliptic to elliptic-oblong, acuminate, long-narrowed below to an articulated case, submembranaceous, plicate, 5- to 7-nerved. Inflorescence up to 52 cm long, arising from the axil of one of the middle leaves; peduncle 43 cm long, dull lavender, glabrous, remotely concealed with six short, tubular, acute sheaths; raceme up to 9 cm long, very loosely few-flowered. Flowers purple, subfleshy, with the sepals projecting backwards and the petals erect. Floral bracts small, narrowly lanceolate, concave, equaling about half of the pedicellate ovary. Ovary up to 23 mm long. Dorsal sepal 15 mm long, 4.4 mm wide, lanceolate-oblong, obtuse to acute. Lateral sepals 16 mm long, 6 mm wide, similar, obliquely lanceolate-oblong, obtuse to acute. Petals 12 mm long, 7 mm wide, distinctly shorter and broader than the sepals. Lip 11 mm long, 10 mm wide, simple, clawed, with involute undulate margins; claw short but distinct, abruptly dilated from an oblong base, 3 mm long, furnished with a central fleshy callus dividing into two branches; lamina gently recurved, triangular-ovate, rounded at the apex, conspicuously cordate at the base, fleshy thickened in the middle. Gynostemium 5 mm long, stout.

Flowering time: April.

Distribution: Venezuela. Elev. 2125–2300 m a.s.l.

## Supporting Information

Table S1 **Variables used in the modelling.**
(DOC)

Table S2 **The average training AUC for the replicate runs (AUC - area under the curve, SD – standard deviation).**
(DOC)

Annex S1 **List of analysed taxa downloaded from International Nucleotide Sequence Databases.** INSD accession numbers for DNA sequences are listed in following order: ITS matK trnL-F.
(DOC)

Annex S2 **Set of the characters used in the phenetic analysis.**
(DOC)

Annex S3 **Morphological character matrix.**
(DOC)

Annex S4 **Localities of the specimens collection of** ***Vargasiella*** **C.Schweinf. and** ***Warrea*** **Lindl. used in the ENM analysis.**
(DOC)
