# Supplementary material for: Taxonomic Position and Phylogeny of the Genus Vargasiella (Orchidaceae, Vandoideae) Based on Molecular and Morphological Evidence
Source: PLoS One. 2014 Jun 3;9(6):e98472. doi: 10.1371/journal.pone.0098472 (PMC4043880; doi:10.1371/journal.pone.0098472)
Supplement: Table S1 — Variables used in the modelling. (DOC) [file pone.0098472.s001.doc]

| Code | Variable |
| --- | --- |
| bio1 | Annual Mean Temperature |
| bio2 | Mean Diurnal Range = Mean of monthly (max temp - min temp) |
| bio3 | Isothermality (bio2/bio7) (* 100) |
| bio4 | Temperature Seasonality (standard deviation *100) |
| bio5 | Max Temperature of Warmest Month |
| bio6 | Min Temperature of Coldest Month |
| bio7 | Temperature Annual Range (bio5-bio6) |
| bio8 | Mean Temperature of Wettest Quarter |
| bio9 | Mean Temperature of Driest Quarter |
| bio10 | Mean Temperature of Warmest Quarter |
| bio11 | Mean Temperature of Coldest Quarter |
| bio12 | Annual Precipitation |
| bio13 | Precipitation of Wettest Month |
| bio14 | Precipitation of Driest Month |
| bio15 | Precipitation Seasonality (Coefficient of Variation) |
| bio16 | Precipitation of Wettest Quarter |
| bio17 | Precipitation of Driest Quarter |
| bio18 | Precipitation of Warmest Quarter |
| bio19 | Precipitation of Coldest Quarter |
| Alt | Altitude |
