# Supplementary material for: Taxonomic Position and Phylogeny of the Genus Vargasiella (Orchidaceae, Vandoideae) Based on Molecular and Morphological Evidence
Source: PLoS One. 2014 Jun 3;9(6):e98472. doi: 10.1371/journal.pone.0098472 (PMC4043880; doi:10.1371/journal.pone.0098472)
Supplement: Table S2 — The average training AUC for the replicate runs (AUC - area under the curve, SD – standard deviation). (DOC) [file pone.0098472.s002.doc]

| **Studied time period** | **Dataset** | **AUC** |
| --- | --- | --- |
| LGM | *Vargasiella* sp. | 0.997 (SD=0.001) |
|  | *Warrea costaricensis* | 0.993 (SD=0.003) |
|  | *Warrea warreana* | 0.975 (SD=0.015) |
|  | *Warreopsis pardina* | 1 (SD=0) |
|  | *Warreopsis parviflora* | 0.999 (SD=0) |
| Present | *Vargasiella* sp. | 0.998 (SD=001) |
|  | *Warrea costaricensis* | 0.992 (SD=0.003) |
|  | *Warrea warreana* | 0.973 (SD=0.017) |
|  | *Warreopsis pardina* | 1 (SD=0) |
|  | *Warreopsis parviflora* | 0.999 (SD=0) |
