# Supplementary material for: Taxonomic Position and Phylogeny of the Genus Vargasiella (Orchidaceae, Vandoideae) Based on Molecular and Morphological Evidence
Source: PLoS One. 2014 Jun 3;9(6):e98472. doi: 10.1371/journal.pone.0098472 (PMC4043880; doi:10.1371/journal.pone.0098472)
Supplement: Annex S1 — List of analysed taxa downloaded from International Nucleotide Sequence Databases. INSD accession numbers for DNA sequences are listed in following order: ITS matK trnL-F. (DOC) [file pone.0098472.s003.doc]

*Acacallis cyanea* Lindl. AY870104 AY870005 AY869907, *Acacallis fimbriata* (Rchb. *f*.) Schltr. Breuer & Gerlach AY870105 AY870006 AY869908, *Ackermania caudata* (Ackerman) Dodson & R.Escobar AY870027 AY869928 AY869842, *Ackermania cornuta* (Garay) Dodson & R. Escobar AY870026 AY869930 AY86984, *Ackermania hajekii* D.E.Benn. & Christenson AY870028 AY869929 AY869843, *Aganisia pulchella* Lindl. AY870106 AY870007 AY869909, *Benzingia estradae* (Dodson) Dodson *ex* Dodson AY870029 AY869930 AY869844, *Bollea ecuadorana* Dodson AY870050 AY869951 AY869863, *Bollea lawrenciana* Rchb.*f*. AY870048 AY869949 AY869861, *Bollea pulvinaris* Rchb.*f*. AY870049 AY869950 AY869862, *Chaubardia klugii* (C. Schweinf.) Garay AY870072 AY869973 AY869885, *Chaubardia surinamensis* Rchb.*f*. AY870073 AY869974 AY869886, *Chaubardiella pacuarensis* Jenny AY870046 AY869947 AY869859, *Chaubardiella pubescens* Ackerman AY870043 AY869944 AY869856, *Chaubardiella subquadrata* (Schltr.) Garay AY870044 AY869945 AY869857, *Chaubardiella tigrina* (Garay & Dunst.) Garay AY870045 AY869946 AY869858, *Chondrorhyncha* aff. *carinata* P. Ortiz AY870040 AY869941 AY869853, *Chondrorhyncha albicans* Rolfe AY870016 AY869917 AY869831, *Chondrorhyncha andreae* P. Ortiz AY870047 AY869948 AY869860, *Chondrorhyncha crassa* Dressler AY870017 AY869918 AY869832, *Chondrorhyncha hirtzii* Dodson AY870015 AY869916 AY869830, *Chondrorhyncha lankesteriana* Pupulin AY869832 AY869962 AY869874, *Chondrorhyncha lendyana* Rchb.*f.* AY870062 AY869963 AY869875, *Chondrorhyncha litensis* Dodson AY870039 AY869940 AY869852, *Chondrorhyncha picta* (Rchb.*f*.) Senghas AY870060 AY869961 AY869873, *Chondrorhyncha viridisepala* Senghas #1 AY870041 AY869942 AY869854, *Chondrorhyncha viridisepala* Senghas #2 AY870042 AY869943 AY869855, *Chondroscaphe* aff. *chestertonii* (Rchb.*f*.) Senghas & G. Gerlach AY870069 AY869970 AY869882, *Chondroscaphe amabilis* (Schltr.) Senghas & G. Gerlach AY870065 AY869966 AY869878, *Chondroscaphe atrilinguis* AY870071 AY869972 AY869884, *Chondroscaphe* cf. *laevis* Dressler AY870067 AY869968 AY869880, *Chondroscaphe eburnea* (Dressler) Dressler AY870014 AY869915 AY869829, *Chondroscaphe escobariana* (Dodson & Neudecker) C. Rungius *ex* C. Rungius AY870066 AY869967 AY869879 *Chondroscaphe flaveola* (Linden & Rchb.*f*. e*x* Rchb.*f*.) Senghas & G. Gerlach AY870068 AY869969 AY869881, *Chondroscaphe laevis* Dressler AY870067 AY869968 AY869883, *Cochleanthes anatona* Dressler AY870032 AY869933 AY869845, *Cochleanthes aromatica* (Rchb.*f*.) R.E. Schultes & Garay AY870063 AY869964 AY869876, *Cochleanthes flabelliformis* (Sw.) R.E. Schultes & Garay AY870064 AY869965 AY869877, *Cochleanthes guianensis* A. Lafontaine, G.Gerlach & K. Senghas AY870055 AY869956 AY869868, *Cochleanthes guianensis* A. Lafontaine, G.Gerlach & K. Senghas AY870056 AY869957 AY869869, *Cochleanthes wailesiana* (Lindl.) R.E. Schult.& Garay AY870059 AY869960 AY869872, *Cryptarrhena guatemalensis* Schltr. F. Pupulin & J.A. Campos AY870082 AY869983 AY869895, *Cryptarrhena lunata* R. Br. AY870081 AY869982 AY869894, *Dichaea campanulata* C. Schweinf. AY870079 AY869980 AY869892, *Dichaea panamensis* Lindl. AY870080 AY869981 AY869893, *Dichaea* aff. *morrisii* Fawc. & Rendle Pupulin AY870078 AY869979 AY869891, *Dichaea squarrosa* Lindl. AY869891 AY869978 AY869890, *Dodsonia saccata* (Garay) Ackerman AY870024 AY869925 AY869839, *Galeottia fimbriata* Linden & Rchb.*f*. AY870091 AY869992 AY869896, *Galeottia grandiflora* A. Rich. AY870092 AY869993 AY869897, *Huntleya gustavii* (Rchb.*f*.) Schltr. AY870076 AY869977 AY869889, *Huntleya wallisii* (Rchb.*f*.) Rolfe #1 AY870074 AY869975 AY869887, *Huntleya wallisii* (Rchb.*f*.) Rolfe #2 AY870075 AY869976 AY869888, *Kefersteinia excentrica* Dressler & Mora-Retana AY870033 AY869934 AY869846, *Kefersteinia expansa* (Rchb.*f*.) Schltr. AY870038 AY869939 AY869851, *Kefersteinia guacamayoana* Dodson & Hirtz AY869935 AY869847, *Kefersteinia maculosa* Dressler AY870037 AY869938 AY869850, *Kefersteinia microcharis* Schltr. AY870036 AY869937 AY869849, *Kefersteinia trullata* Dressler Whitten 1998 FLAS AY870035 AY869936 AY869848, *Koellensteinia graminea* (Lindl.) Rchb.*f* AY870102 AY870003 AY869906, *Maxillaria violaceopunctata* Rchb.*f*. AY870109 AY870010 AY869911, *Neogardneria murrayana* (Gardner *ex* Hook.) Schltr. AY870096 AY869997 AY869900, *Pabstia jugosa* (Lindl.) Garay AY870098 AY869999 AY869902, *Paradisanthus micranthus* (Barb. Rodr.) Schltr. AY870107 AY870008 AY869910, *Pescatorea cerina* (Lindl. & Paxton) Rchb.*f*. AY870051 AY869952 AY869864, *Pescatorea coronaria* Rchb.*f*. AY870053 AY869954 AY869866, *Pescatorea lamellosa* Rchb.*f*. AY870052 AY869953 AY869865, *Promenaea ovatiloba* (Klinge) Cogn. AY870100 AY870001 AY869904, *Promenaea stapelioides* (Link & Otto) Lindl. AY870101 AY870002 AY869905, *Promenaea xanthina* Lindl. AY870099 AY870000 AY869903, *Rudolfiella saxicola* (Schltr.) C. Schweinf. AY870110 AY870011 AY869912, *Stenia* aff*. wendiae* D.E. Benn. & Christenson AY870023 AY869924 AY869838, *Stenia bismarckii* Dodson & D.E. Benn*.* AY870019 AY869920 AY869834, *Stenia calceolaris* (Garay) Dodson & D.E. Benn. AY870018 AY869919 AY869833, *Stenia glatzii* Neudecker & Gerlach AY870020 AY869921 AY869835, *Stenia pallida* Lindl. AY870021 AY869922 AY869836, *Stenia pallida* Lindl. AY870022 AY869923 AY869837, *Warczewiczella wailesiana* (Lindl.) Rchb.*f. ex* E. Morren AY870059 AY869960 AY869872, *Warczewiczella discolor* (Lindl.) Rchb.*f*. AY870058 AY869959 AY869871, *Warczewiczella lipscombiae* (Rolfe) Fowlie AY870054 AY869955 AY869867, *Warczewiczella marginata* Rchb.f. AY870057 AY869958 AY869870, *Warrea warreana* (Lodd. *ex* Lindl.) C. Schweinf AF239321 AF239417 AF239513, *Warreopsis colorata* (Linden & Rchb.*f*.) Garay AY870083 AY869984 - , *Warreopsis pardina* (Rchb.f.) Garay AY870084 AY869985 - , *Zygopetalum intermedium* Lodd. *ex*. Lindl. AY870097 AY869998 AY869901, *Zygopetalum maxillare* Lodd. AY870095 AY869996 AY869899, *Zygosepalum labiosum* (Rich.) Garay AY870094 AY869995 AY869898
