# Supplementary material for: Taxonomic Position and Phylogeny of the Genus Vargasiella (Orchidaceae, Vandoideae) Based on Molecular and Morphological Evidence
Source: PLoS One. 2014 Jun 3;9(6):e98472. doi: 10.1371/journal.pone.0098472 (PMC4043880; doi:10.1371/journal.pone.0098472)
Supplement: Annex S2 — Set of the characters used in the phenetic analysis. (DOC) [file pone.0098472.s004.doc]

**1) Pseudobulbs:**

0 – absent; 1 – homoblastic; 2 – heteroblastic

**2) Leaves:**

0 – convolute; 1 - conduplicate

**3) Inflorescence:**

0 – subapical; 1 – subbasal or basal

**4) Lip:**

0 – simple; 1 - variously lobed

**5) Lip callus**

0 – obscure; 1 - prominent, flabellate or simple; 2 - tabula infrastigmatica

**6) Gynostemium:**

0 - erect, rather short; 1 - elongate

**7) Colum foot:**

0 – absent; 1 – obscure, short; 2 – prominent

**8) Caudicles:**

0 - sticky, amorphous; 1 - ribbon-like

**9) Anther:**

0 – ventral; 1 - subapical

**10) Pollinia:**

0 - 4 in two pairs; 1 - 2, subglobose

**11) Clinandrium:**

0 – obscure; 1 – spacious

**12) Viscidium:**

0 - elliptic-cordate to hippocrepiform; 1 - single, elliptic-ovate, lamellar; 2 - single, oblong elliptic, thick, fleshy

**13) Rostellum:**

0 – short, broad; 1 - rather large, dome-like

**14) Rostellum remnant:**

0 - with short apiculus in the middle; 1 - 3-lobed; 2 - canaliculated, bilobulate at the apex.
