# Supplementary material for: Taxonomic Position and Phylogeny of the Genus Vargasiella (Orchidaceae, Vandoideae) Based on Molecular and Morphological Evidence
Source: PLoS One. 2014 Jun 3;9(6):e98472. doi: 10.1371/journal.pone.0098472 (PMC4043880; doi:10.1371/journal.pone.0098472)
Supplement: Annex S3 — Morphological character matrix. (DOC) [file pone.0098472.s005.doc]

| **Character** | ***Vargasiella*** | ***Warrea*** | ***Warreopsis*** | ***Maxillaria*** | ***Zygopetalum*** | ***Cryptarrhena*** | ***Oncidium*** |
| --- | --- | --- | --- | --- | --- | --- | --- |
| Pseudobulbs | 0 | 1 | 1 | 2 | 2 | 2 | 2 |
| Leaves | 0 | 0 | 0 | 1 | 0 | 1 | 1 |
| Inflorescence | 0 | 1 | 1 | 1 | 1 | 1 | 1 |
| Inflorescence | 0 | 0 | 0 | 1 | 0 | 0 | 0 |
| Lip | 0 | 0 | 0 | 1 | 0 | 1 | 1 |
| Callus | 0 | 1 | 1 | 1 | 1 | 0 | 2 |
| Gynostemium | 0 | 1 | 1 | 1 | 1 | 0 | 1 |
| Column foot | 1 | 1 | 1 | 2 | 2 | 0 | 0 |
| Caudicles | 0 | 0 | 0 | 0 | 0 | 1 | 0 |
| Anther | 0 | 0 | 0 | 1 | 0 | 1 | 1 |
| Pollinia | 0 | 0 | 0 | 0 | 0 | 0 | 1 |
| C;linandrium | 0 | 0 | 0 | 0 | 0 | 1 | 0 |
| Viscidium | 0 | 1 | 1 | 0 | 1 | 2 | 2 |
| Rostellum | 0 | 1 | 1 | 1 | 1 | 0 | 0 |
| Rostellum remnant | 0 | 1 | 1 | 2 | 1 | 2 | 2 |
