# Supplementary material for: Taxonomic Position and Phylogeny of the Genus Vargasiella (Orchidaceae, Vandoideae) Based on Molecular and Morphological Evidence
Source: PLoS One. 2014 Jun 3;9(6):e98472. doi: 10.1371/journal.pone.0098472 (PMC4043880; doi:10.1371/journal.pone.0098472)
Supplement: Annex S4 — Localities of the specimens collection of Vargasiella C.Schweinf. and Warrea Lindl. used in the ENM analysis. (DOC) [file pone.0098472.s006.doc]

| **Species** | **Country** | **Latitude** | **Longitude** | **Collector(s) and number** | **Herbarium acronym** |
| --- | --- | --- | --- | --- | --- |
| *Vargasiella peruviana* | Bolivia | -16,3 | -67,8 | Solomon 14982 | LPB, MO |
| *Vargasiella peruviana* | Peru | -10,5833 | -75,25 | Gentry & Smith 35984 | MO |
| *Vargasiella peruviana* | Peru | -10,2994 | -75,61 | Valenzuela & al. 13671 | MO, MOL, USM |
| *Vargasiella peruviana* | Peru | -10,5833 | -75,3333 | Foster & Smith 9094 | MO |
| *Vargasiella peruviana* | Peru | -10,65 | -75,5667 | van der Werff & al. 8508 | MO |
| *Vargasiella venezuelana* | Venezuela | 5,225297 | -62,2328 | Steyermark 74914 | AMES |
| *Warrea warreana* | Argentina | -26,9166 | -54,2166 | Zuloaga & Morrone 6898 | SI |
| *Warrea warreana* | Bolivia | -16,3833 | -63,4583 | Vásquez & al.. 225 | Herb. Vasquez, LPB |
| *Warrea warreana* | Ecuador | -1,85 | -78 | Dodson & al. 17787 | QCNE, RPSC |
| *Warrea warreana* | Colombia | 3,59 | -76,8291 | Szlachetko 9188 | UGDA |
| *Warrea warreana* | Ecuador | -0,2666 | -78,9986 | Dodson & al. 10553 | SEL |
| *Warrea costaricensis* | Costa Rica | 9,83 | -84,01 | Lankester 1761 | SEL |
| *Warrea costaricensis* | Costa Rica | 9,85 | -83,69 | Dodson 2534 | SEL |
| *Warrea costaricensis* | Costa Rica | 10,2 | -84,53 | Grayum & al. 6338 | MO |
| *Warrea costaricensis* | Costa Rica | 10,31 | -84,72 | Bello C. 5180 | CR |
| *Warrea costaricensis* | Costa Rica | 10,4313 | -84,0036 | Hammel 10161 | DUKE |
| *Warrea costaricensis* | Costa Rica | 10,42 | -84,02 | Todzia 1214 | MO |
| *Warrea costaricensis* | Costa Rica | 8,6569 | -83,1569 | Chavarría & al. 537 | MO |
| *Warrea costaricensis* | Costa Rica | 8,81 | -83,19 | Hammel & al. 19249 | CR |
| *Warrea costaricensis* | Costa Rica | 8,74 | -83,56 | Herrera Ch. & al. 4607 | CR |
| *Warrea costaricensis* | Guatemala | 16,5394 | -89,3788 | Contreras 3191 | LL |
| *Warrea costaricensis* | Honduras | 15,7 | -87,4666 | MacDougal & al. 3354 | MO |
| *Warrea costaricensis* | Mexico | 16,76 | -91 | Martínez S. 15631 | MO |
| *Warrea costaricensis* | Nicaragua | 14,0333 | -83,3833 | Heller 8873 | MO |
| *Warrea costaricensis* | Panama | 9,2027 | -79,3916 | Dressler 3250 | MO |
| *Warreopsis parviflora* | Costa Rica | 10,3333 | -84,8333 | Haber & al. 4711 | MO |
| *Warreopsis parviflora* | Costa Rica | 10,12 | -84,55 | Brenes 1637 | F |
| *Warreopsis parviflora* | Costa Rica | 10,09 | -84,06 | Lent 2624 | F |
| *Warreopsis parviflora* | Costa Rica | 10,05 | -84,0267 | Davidse & Pohl 1692A | MO |
| *Warreopsis parviflora* | Panama | 8,85 | -82,5167 | Woodson, Jr. & Schery 605 | MO |
| *Warreopsis pardina* | Ecuador | - 78,4983 | -0,10886 | Hirtz 201 | RPSC |
| *Warreopsis pardina* | Ecuador | - 78,8881 | -0,25 | Dodson & al. 10553 | SEL |
| *Warreopsis pardina* | Ecuador | -78,7 | -0,04 | Dalström 1603 | SEL |
| *Warreopsis pardina* | Ecuador | - 78,8 | -0,0333 | Jameson s.n. | AMES |
| *Warreopsis pardina* | Ecuador | - 78,8167 | -0,2333 | Molau & al. 3017 | MO database |
